# Progress in Continuous Flow Synthesis of Hydrogen-Bonded Organic Framework Material Synthons

**DOI:** 10.3390/molecules30010041

**Published:** 2024-12-26

**Authors:** Xingjun Yao, Sanmiao Wen, Ningning Ji, Qiulin Deng, Zhiliang Li, Hongbing Wang, Qianqian Shang

**Affiliations:** 1 Shandong Provincial Key Laboratory of Chemical Energy Storage and Novel Cell Technology, School of Chemistry and Chemical Engineering, Liaocheng University, Liaocheng 252059, China; 2 College of Chemistry and Chemical Engineering, Taishan University, Tai’an 271021, China; 3 School of Materials Science and Engineering, Southwest University of Science and Technology, Mianyang 621010, China; 4 School of Materials Science and Engineering, Liaocheng University, Liaocheng 252059, China

**Keywords:** hydrogen-bonded organic frameworks (HOFs), synthons, continuous flow, microreactor, microchannel

## Abstract

Hydrogen-bonded organic framework (HOF) materials are typically formed by the self-assembly of small organic units (synthons) with specific functional groups through hydrogen bonding or other interactions. HOF is commonly used as an electrolyte for batteries. Well-designed HOF materials can enhance the proton exchange rate, thereby boosting battery performance. This paper reviews recent advancements in the continuous synthesis of HOF synthons, in the continuous synthesis of HOF’s unit small molecules enabling the multi-step, rapid, and in situ synthesis of synthons, such as carboxylic acid, diaminotriazine (DAT), urea, guanidine, imidazole, pyrazole, pyridine, thiazole, triazole, and tetrazole, with online monitoring. Continuous flow reactors facilitate fast chemical reactions and precise microfluidic control, offering superior reaction speed, product yield, and selectivity compared to batch processes. Integrating the continuous synthesis of synthons with the construction of HOF materials on a single platform is essential for achieving low-cost, safe, and efficient processing, especially for reactions involving toxic, flammable, or explosive substances.

## 1. Introduction

Hydrogen-bonded organic framework (HOF) materials are formed by the self-assembly of rigid organic structural units containing specific functional groups under controlled conditions such as temperature, solvent polarity, and reaction time. These functional groups, typically containing nitrogen and oxygen, facilitate hydrogen bonding, which is essential for building stable frameworks.

HOF materials offer several unique advantages: (1) Their synthesis conditions are generally milder compared to other frameworks like Covalent organic framework materials (COFs) or Metal-organic framework materials (MOFs), and they are easier to process in solution, making them suitable for device fabrication [1]. For example, Feng, S. et al. successfully prepared UPC-HOF-6 thin films using solution processing, which showed potential in pressure-responsive H_2_/N_2_ separation [2]. (2) HOFs possess remarkable self-healing and regenerative capabilities. Hu, F. et al. developed a highly stable, high-surface-area HOF (HOF-TCBP) that can be regenerated with minimal solvent treatment, retaining nearly the same surface area as the original material [3]. (3) Due to the absence of metal ions, HOFs demonstrate excellent biocompatibility and low cytotoxicity, making them promising for applications in fluorescence sensing, biomedicine, and membrane technology.

Recent studies show that HOFs, with their stable microporous structures and well-defined hydrogen-bonded networks, serve as effective solid-state proton conductors, achieving conductivities up to 10^−1^ s·cm^−1^. This makes them suitable for use as solid electrolytes in fuel cells and other electrochemical devices [4]. Additionally, HOFs can act as precursor templates for high-temperature annealing into metal compounds, carbon materials, or composites, offering nanostructural benefits for energy storage applications [5].

Structurally, HOFs are crystalline porous materials built from organic molecules interconnected by hydrogen bonds, with additional stabilization provided by π-π stacking, electrostatic interactions, and van der Waals forces [6]. Stability can be further enhanced by selecting organic groups capable of forming multiple hydrogen bonds, increasing the number of benzene rings in structural units, using low-polarity solvents, and incorporating interpenetration or interlocking elements [7,8,9,10,11].

The concept of supramolecular synthons, which are spatially arranged intermolecular interactions, plays a central role in the design of HOFs [12]. By carefully selecting synthetic subunits with multiple hydrogen bonds and rigid scaffolds, HOFs can achieve specific structures and functionalities. For instance, supramolecular synthons like carboxy, DAT, pyrazolyl and their dimers, trimers, etc., provide structural stability and define the extension direction of hydrogen-bond networks, thereby influencing the porosity and functional properties of HOFs [13,14].

The building blocks of HOFs, often heterocycles and their hydrogen bond structure, are also valuable in pharmaceutical and agricultural chemistry due to their presence in many biologically active molecules [15,16,17]. Notably, 59% of Food and Drug Administration (FDA)-approved drugs contain nitrogen heterocycles, highlighting the need for efficient and sustainable synthesis methods [18]. While traditional microwave-assisted or ultrasound synthesis methods are effective, they are time-consuming and costly for laboratory-scale production [19,20,21]. Recent developments in continuous flow chemistry using homogeneous transition metal catalysts have shown promise, achieving higher activity, increased turnover numbers (TON), a broader substrate range, and improved catalyst reusability [22,23,24,25].

**Figure 1:**
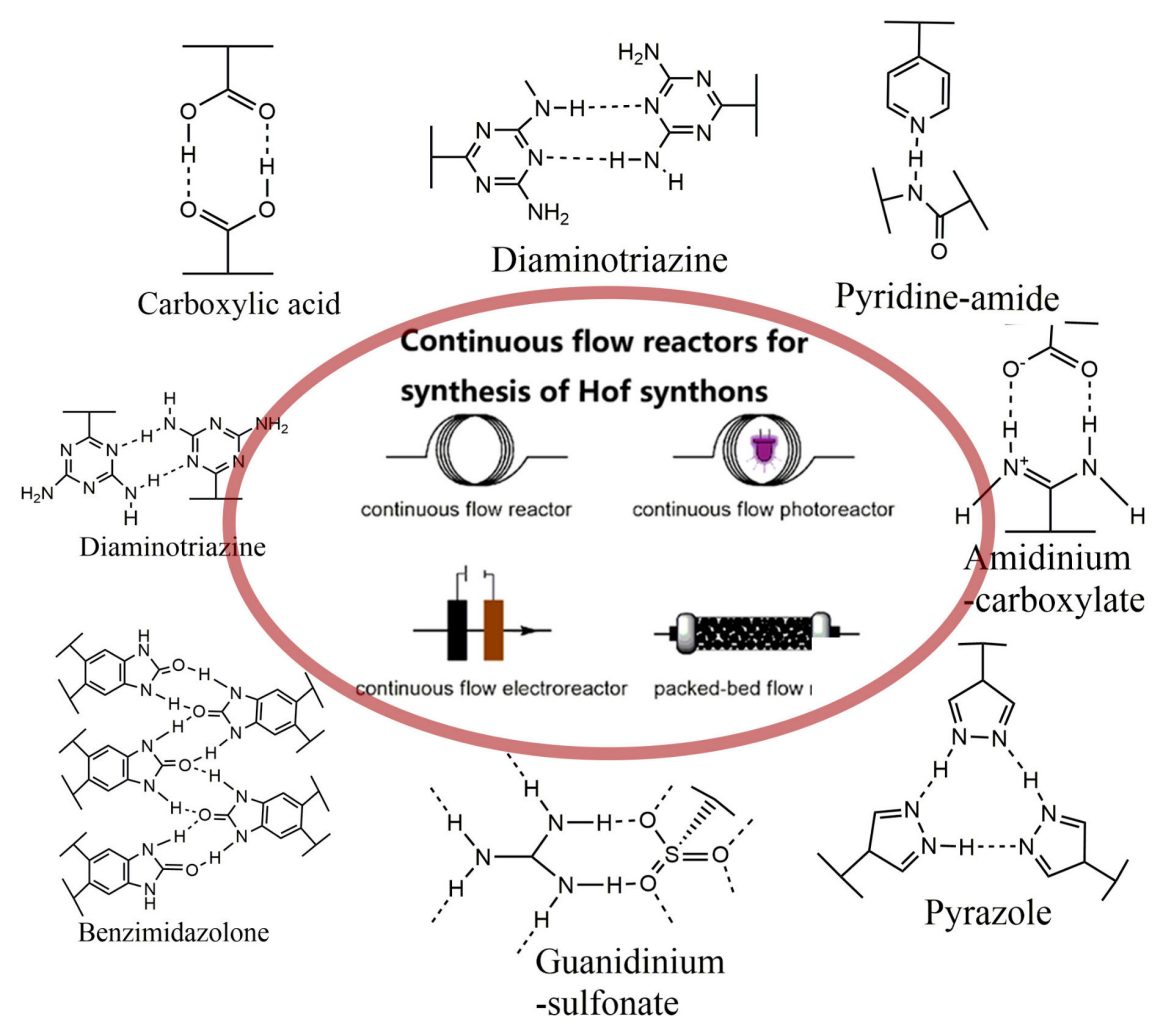
Continuous flow reactor for synthesis of Hof synthons and common hydrogen bonding connection methods for synthons: carboxyl group; diaminotriazine; pyridine; carboxyl and pyridine; amidinium and carboxylate; benzimidazolone; sulfonic acid group and guanidine group; pyrazole.

Continuous-flow chemistry has transformed organic synthesis by offering enhanced safety, efficiency, and waste reduction. Through cascade reactors and online analysis, this approach streamlines multi-step processes, enables real-time data collection, and allows self-optimization, making it a powerful tool for drug discovery and database development [26]. Integrating continuous flow platforms with online analysis and advanced modeling not only improves the understanding of reaction mechanisms but also promotes sustainable and efficient synthesis. This paper reviews recent progress in the continuous synthesis of HOF synthons—such as carboxylic acids, DAT, urea, guanidines, imidazole, pyrazoles, thiazole, pyridine, triazole, and tetrazole-over the past decade (Figure 1). This article is divided into the following parts: 1-Background; 2-Continuous synthesis of HOF synthons; 3-Conclusion.

## 2. Continuous Synthesis of HOF Synthons

### 2.1. Continuous Synthesis of Carboxylic Acids

Carboxylic acids are among the most used motifs in HOF materials. The carboxylate group readily forms O–H···O hydrogen bonds that are highly directional and predictable, making them ideal for constructing porous HOF structures. Notably, the carboxylic acid dimer is a common supramolecular synthon, providing both strength and directionality essential for stable frameworks. The role of hydrophilic carboxyl groups in proton conduction has garnered significant interest, as these groups not only supply protons but also form hydrogen bonds with guest water molecules, facilitating proton transfer [27]. Extensive research has explored HOF proton conduction in carboxylate structures of various types, and numerous studies have reported efficient continuous-flow syntheses of aliphatic, aromatic, and ferrocene carboxylic acid synthons [28,29,30].

An effective and scalable method for transforming alcohols and aldehydes into carboxylic acids in continuous flow has been demonstrated [31]. For example, microreactors are effectively used to oxidize alcohols and aldehydes into carboxylic acids, with homogeneous permanganate (KMnO_4_), serving as an affordable oxidant. Ultrasound was applied to prevent blockages in the capillary reactor caused by MnO_2_ solid formation. The process achieved high product yields (58–98%) and excellent purity (>95%) without requiring chromatography for isolation. The main problem is the reuse of MnO_2_ to minimize environmental impact.

**Figure 2:**
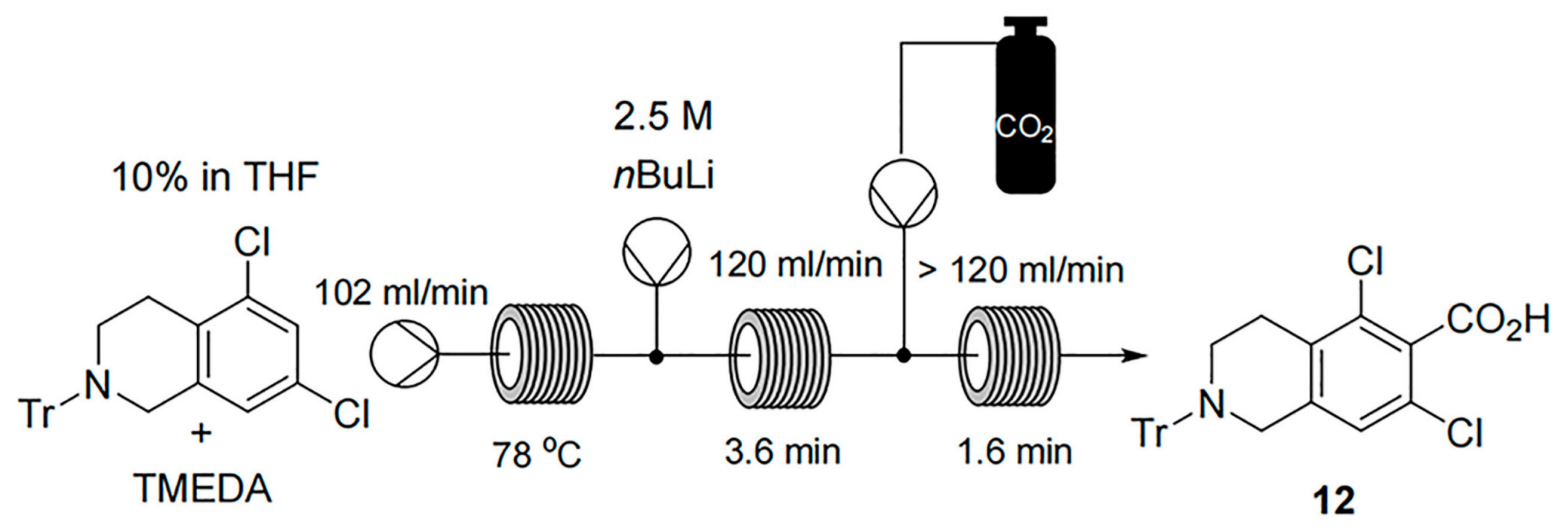
Continuous flow carboxylation yields a central fragment in the synthesis of Lifitegrast [33].

In another example, for the continuous carboxylation step of one of the three fragments which were coupled via an amide bond. The active pharmaceutical ingredient Lifitegrast was synthesized at a low temperature (−78 °C) in a short residence time (3.6 min) [32]. Increasing the concentration of the starting material to 10% improved conversion rates upon scale-up. Reaction with CO_2_ in a subsequent reactor required only 1.6 min for complete conversion. The optimized process consistently yielded 88–91% of the product with a purity of 97–98% over multiple runs, enabling the production of 22 kg of carboxylated product with high yield, reproducibility, and purity at scale (Figure 2) [33].

**Figure 3:**
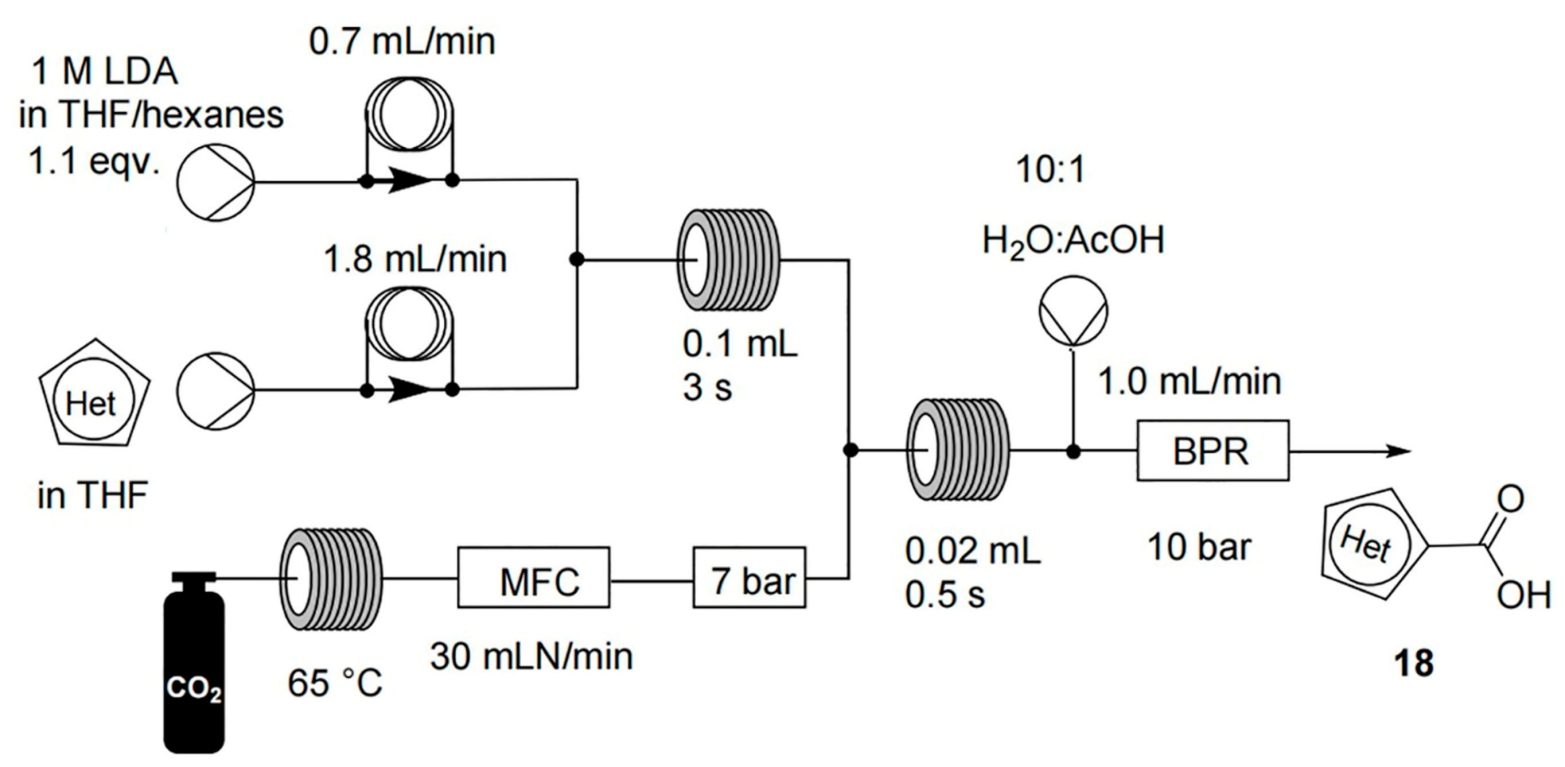
LDA-mediated carboxylation of heterocycles in flow (Adapted from Ref. [34]).

Kappe’s team reported a direct lithiation method for terminal alkynes and heterocycles, followed by carboxylation [34]. This approach utilized two organolithium bases, LiHMDS and LDA, with LDA effectively converting heterocyclic substrates into carboxylic acids. In the designed flow system, heterocyclic substrates were first deprotonated by LDA within a 3 s residence time, generating an anion that then reacted with CO_2_ introduced at a rate of 30 mL/min. Carboxylation occurred in 0.5 s, and the reaction was quenched by a water–acetic acid mixture (10:1) to prevent clogging, which occurred with water alone. This method yielded six heterocyclic carboxylates with yields ranging from 43–79% (Figure 3). Increasing LDA equivalents did not enhance yield, but raising the CO_2_ equivalents to 1.9 slightly improved the yield, demonstrating the system’s robustness and effectiveness for carboxylate synthesis. The exothermic properties of reactions involving organic lithium compounds in microreactors are better controlled, and safety is improved by reducing the risk of combustion and explosion; microreactor technology provides a rapid and effective method for the synthesis of carboxylic acids. CO_2_ is used as a building block and is attractive because it meets the goal of carbon neutrality policy, which is also beneficial for industrial applications.

### 2.2. Continuous Synthesis of Urea

Urea and its derivatives play an essential role in medicinal chemistry, often incorporated into drugs to enhance activity, selectivity, and ADME properties. Due to the delocalization of nitrogen’s non-bonded electrons into adjacent carbonyl groups, the urea group has conformational restrictions that enable it to act as both a hydrogen bond donor and acceptor. This dual property is crucial for regulating drug solubility and permeability by disrupting planarity and modifying interactions with aromatic rings [35,36,37]. The double-stranded H-bond chains formed by urea and thiourea are also used to assemble HOFs [38].

**Figure 4:**
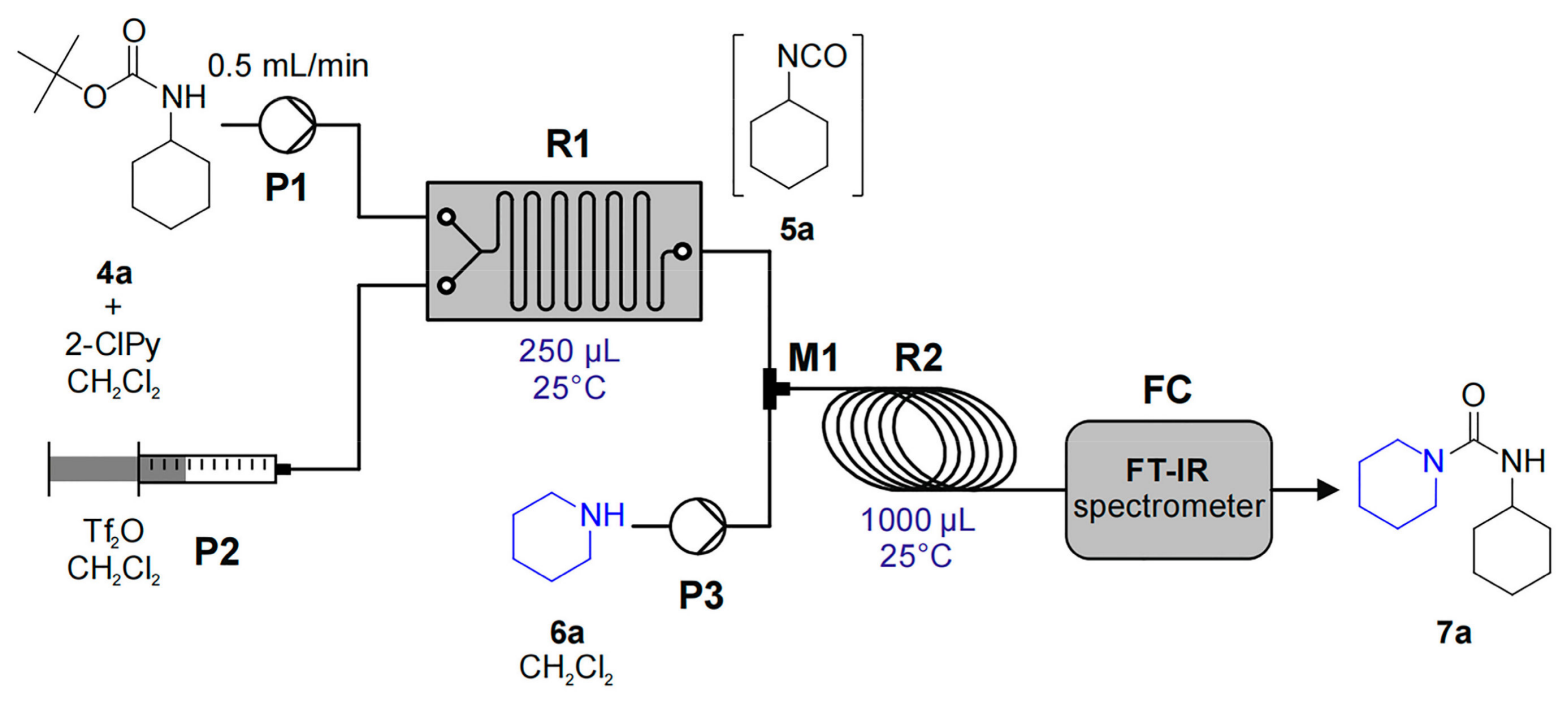
Reactions and flow system consisting of two microreactors and in-line FT-IR spectrometer. Reprinted with permission from Ref. [39]. Copyright 2017 Copyright American Chemical Society.

A two-step continuous-flow system using sequential microreactors has been developed for synthesizing non-symmetrically substituted ureas from protected amines. Under mild conditions, rapid reaction times were achieved, and in-line FT-IR analysis allowed real-time monitoring and optimization of the isocyanate intermediate formation. This setup enabled the synthesis of various urea derivatives, including cariprazine, with the added benefit of minimizing the handling of unstable intermediates (Figure 4) [39]. 

**Figure 5:**
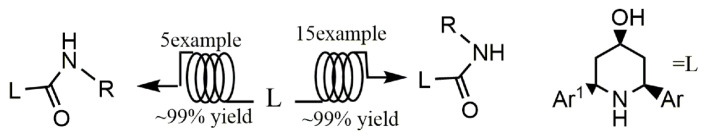
Two libraries of urea compounds based on the piperidine-4-one scaffold have been developed in continuous flow and twenty drug-like compounds were synthesized in moderate to high yields [40].

Riesco-Domínguez, A. et al. expanded on this by synthesizing a library of ureas in a continuous flow setup, achieving drug-like molecules with a reaction time of 17 min and eliminating the need for additional purification steps (Figure 5). Furthermore, they selectively reduced ketones in flow within 3 min and synthesized five urea derivatives from amino alcohols. NMR analysis revealed the presence of rotamers or atropisomers due to restricted rotation around the N-CO bond [40].

**Figure 6:**
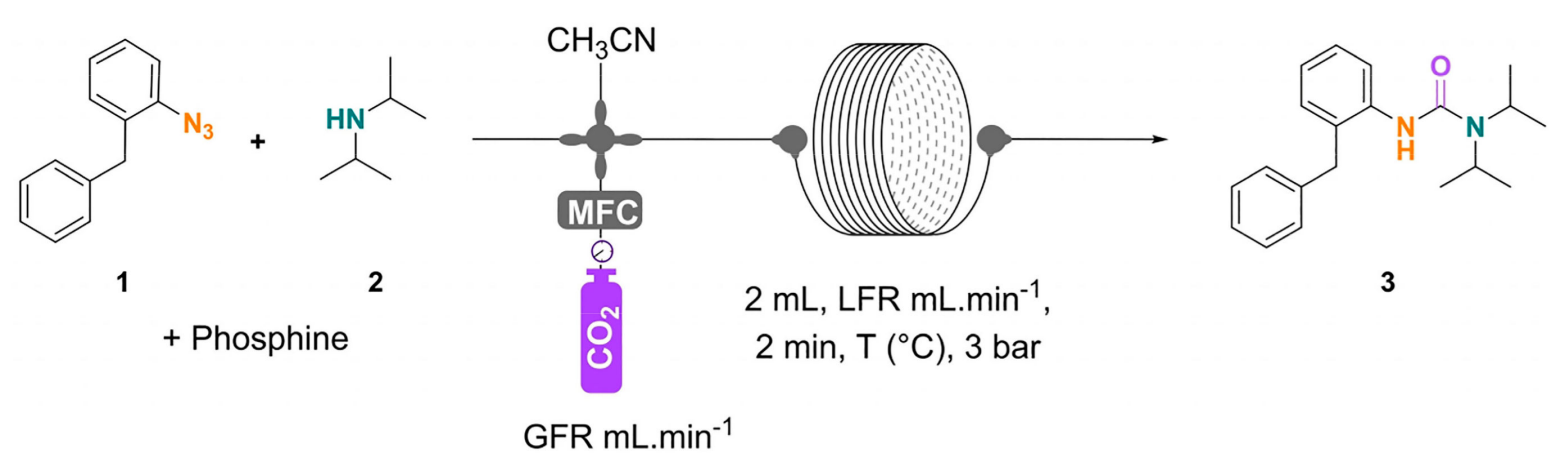

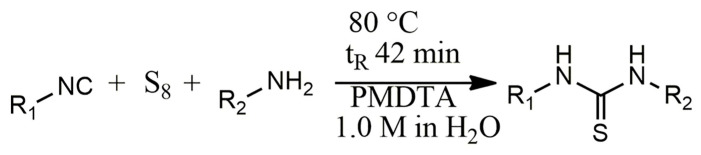
The synthesis of urea and thioureas in continuous flow (Reprinted from Refs. [41,42]).

Labiche, A. et al. implemented a continuous flow synthesis for non-symmetrical ureas via a Staudinger/aza-Wittig reaction, directly obtaining the urea functionality from CO_2_ as the starting material (Figure 6). They synthesized a library of 26 derivatives and extended the methodology to S-thiocarbamates. Meanwhile, Németh et al. developed a continuous-flow process for thioureas by reacting isocyanides, amidines, amines, and sulfur, achieving mild reaction conditions and straightforward product isolation [41,42]. 

Pagano, N. et al. introduced a continuous-flow electrolyzer that utilizes gas diffusion electrodes to catalyze the reduction of CO_2_ and nitrate, producing oxalic acid and urea at the anode and cathode, respectively. Through experimental and theoretical studies, they demonstrated that Pd sites aid in proton hydrogenation of CO_2_ and NO_3_^−^, while Cu and Bi sites stabilize COOH intermediates, promoting C-N bond formation at the cathode [43].

**Figure 7:**
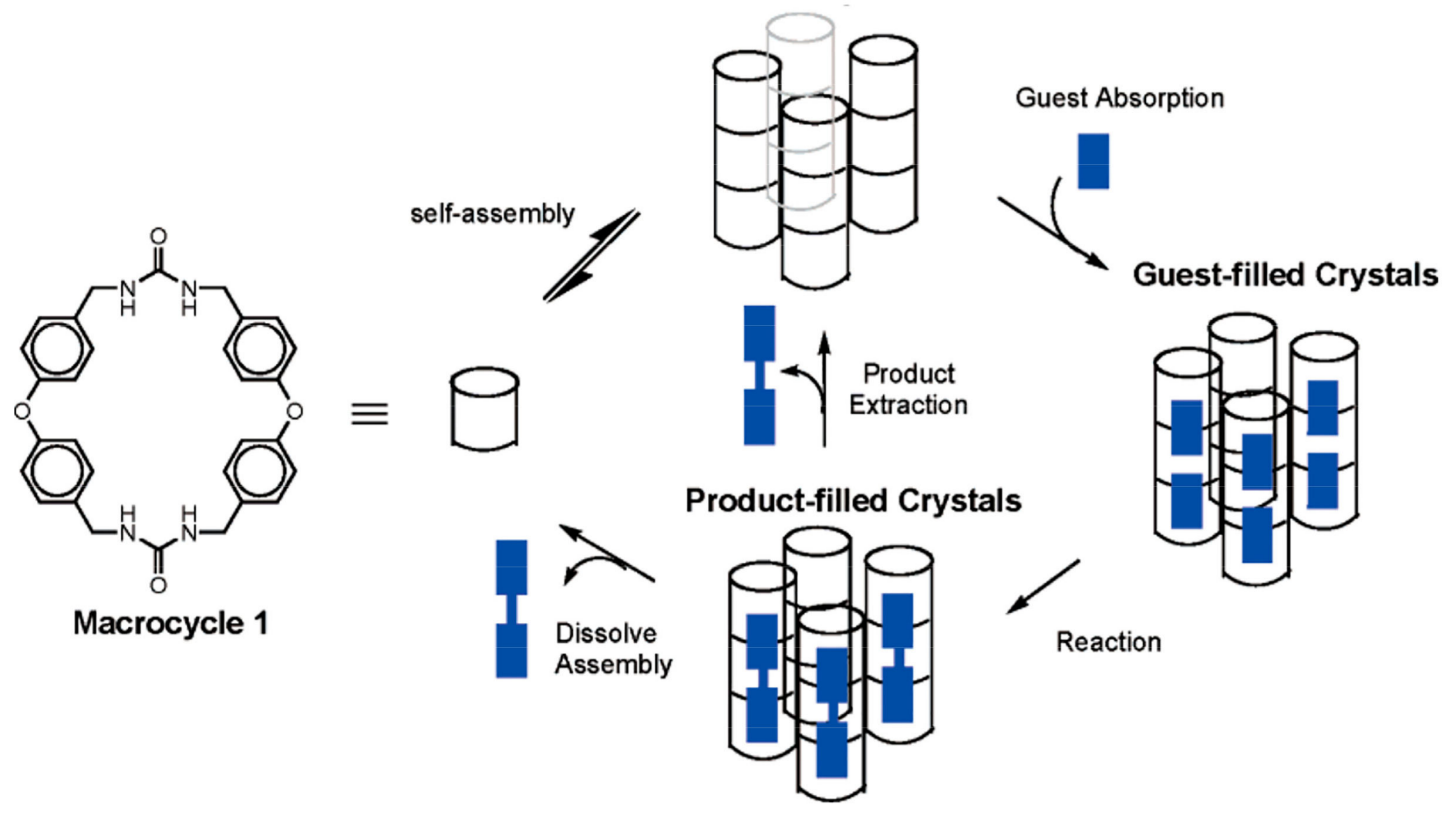
Schematic representation of the self-assembly of macrocycle 1 into crystalline host 1, containing columnar channels. The porous crystals can reversibly absorb a variety of guests including 2-cyclohexenone. UV-irradiation of included guests yields a photo dimer in high conversion and selectivity. Reprinted with permission from Ref. [44]. Copyright 2008 American Chemical Society.

Urea and thiourea derivatives are also used as building blocks in HOFs. For example, incorporating urea moieties within a triptycene skeleton resulted in a complex structure suitable for HOF construction through one-dimensional double hydrogen bonding (Figure 7). Yang, J. et al. explored the selective [2 + 2] cycloadditions of α,β-unsaturated ketones within a bis-urea host, which forms stable inclusion complexes due to accessible channels of approximately 6 Å in diameter [44].

The above similar studies show the specific methodologies, reagents, types of urea derivatives synthesized, yields, purity, target applications, and mechanistic insights. These differences reflect the diverse approaches researchers take to optimize the synthesis of urea derivatives for various applications in medicinal chemistry pharmacological properties such as potency, selectivity, and interactions with biological targets and other fields. The safety of the reaction and the innovation of the synthesis strategy have been realized.

### 2.3. Continuous Synthesis of Diaminotriazine (DAT)

Covalent triazine frameworks (CTFs) are organic polymers formed from 1,3,5-triazine rings with π conjugation. These frameworks exhibit large surface areas with nitrogen atoms that serve as molecular anchoring sites, making CTFs highly effective for gas adsorption and CO_2_ capture. Additionally, CTFs possess micropores, high heat, and chemical stability, making them suitable as catalyst supports and supercapacitor electrode materials [45].

**Figure 8:**
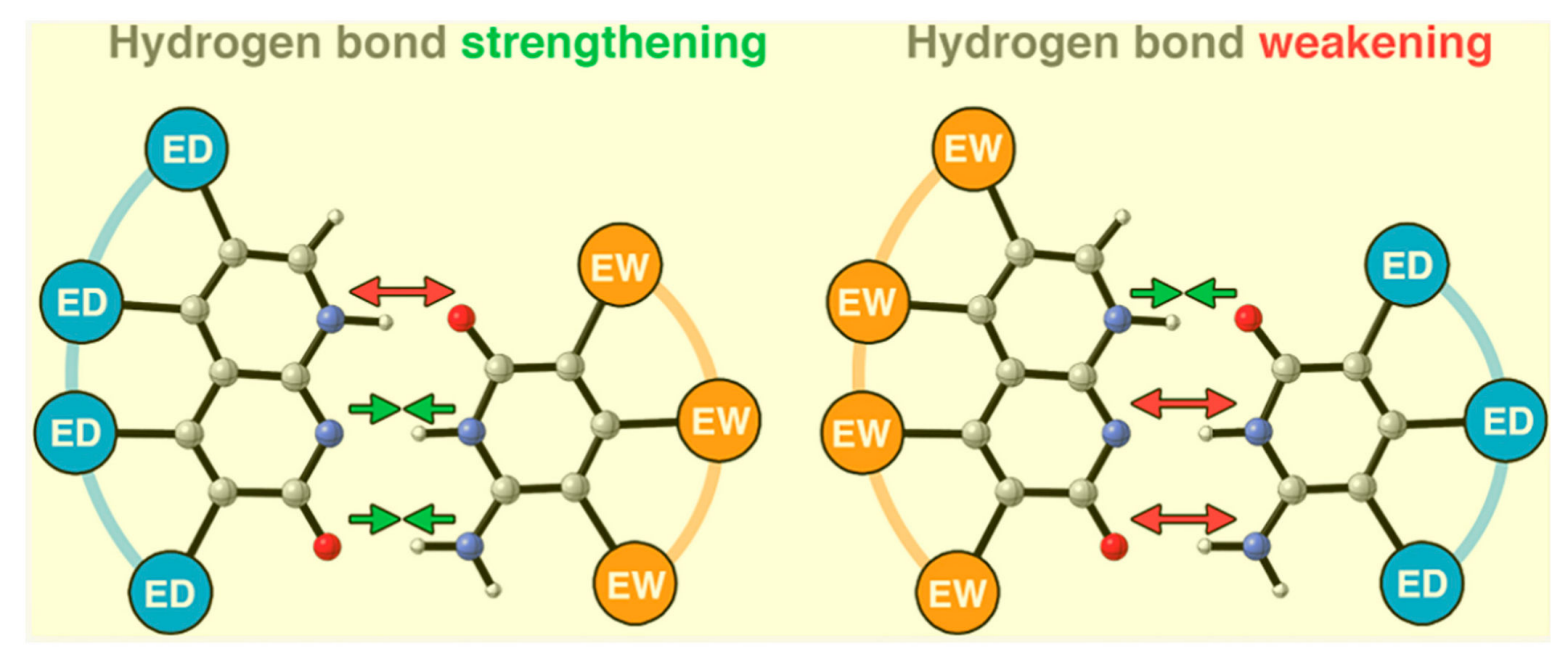
Tuning the binding strength of even and uneven hydrogen-bonded arrays with remote substituents (Reprinted from Ref. [46]). ED (electron-donating group), EW (electron-withdrawing group).

Regulating the strength of hydrogen bonds by changing the charge accumulation around the remote substituents, it is found that the effect of the substituent is delocalized throughout the molecular system; systems with an equal number of hydrogen bond donor (D) and acceptor (A) atoms cannot be regulated in a predictable manner due to the cancellation of opposite effects; the position and long-range electrostatic forces of the substituent also play an important role; the proposed design principles are applicable to monomers with an unequal number of donor and acceptor atoms, and can be used to regulate the binding strength of supramolecular building blocks. (Figure 8) [46]. Through the careful design of H-bonds and coordination interactions of 1,3,5-triazines, various functional materials have been generated [47].

**Figure 9:**
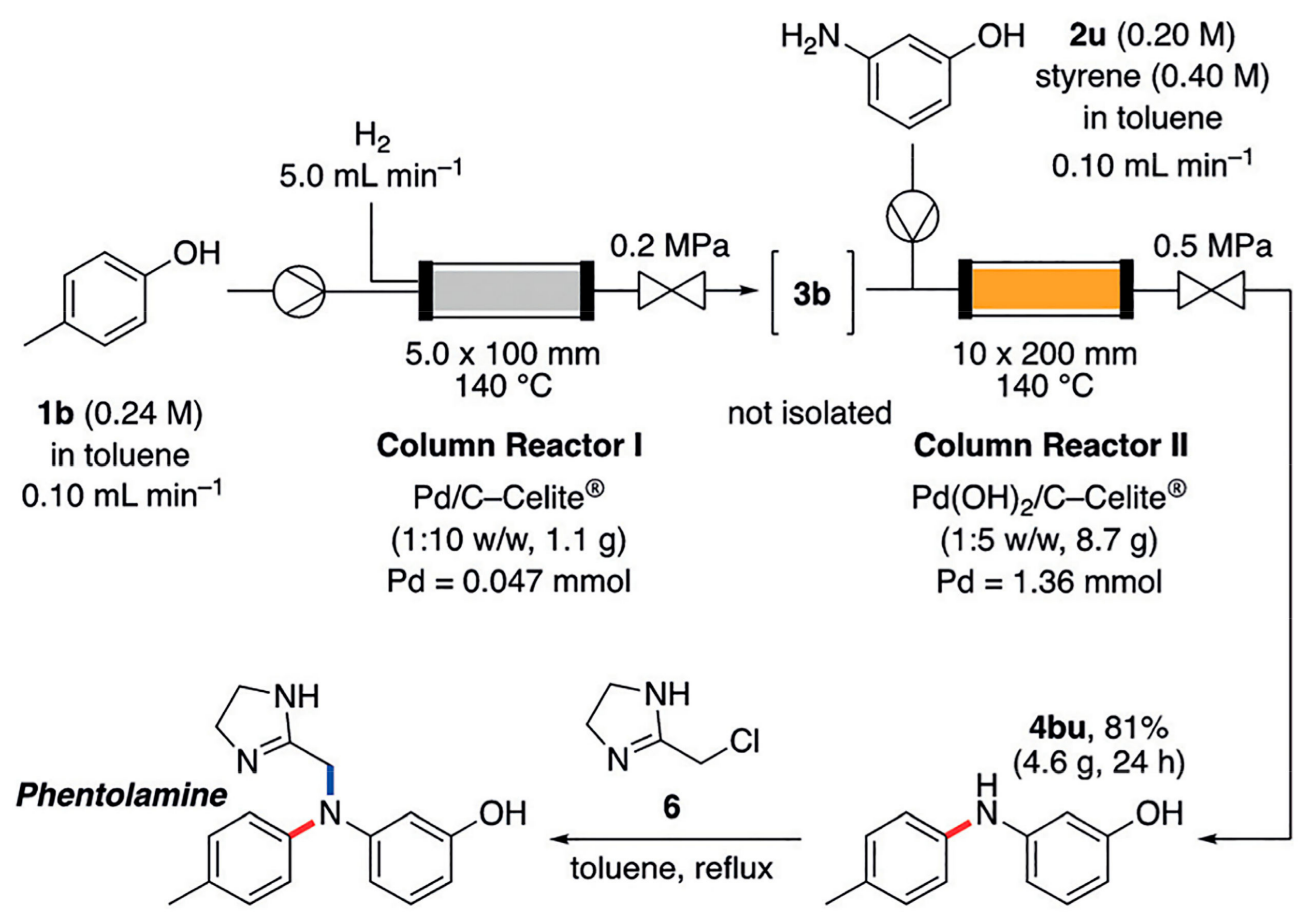
Formal continuous-flow synthesis of phentolamine. Reprinted with permission from Ref. [48]. Copyright 2020 Copyright John Wiley and Sons.

Kobayashi, S. and his team further expanded this process by developing a column reactor system that continuously converts phenols to cyclohexanone derivatives, facilitating high-frequency turnovers and yielding aromatic amines efficiently (Figure 9) [48]. 

**Figure 10:**
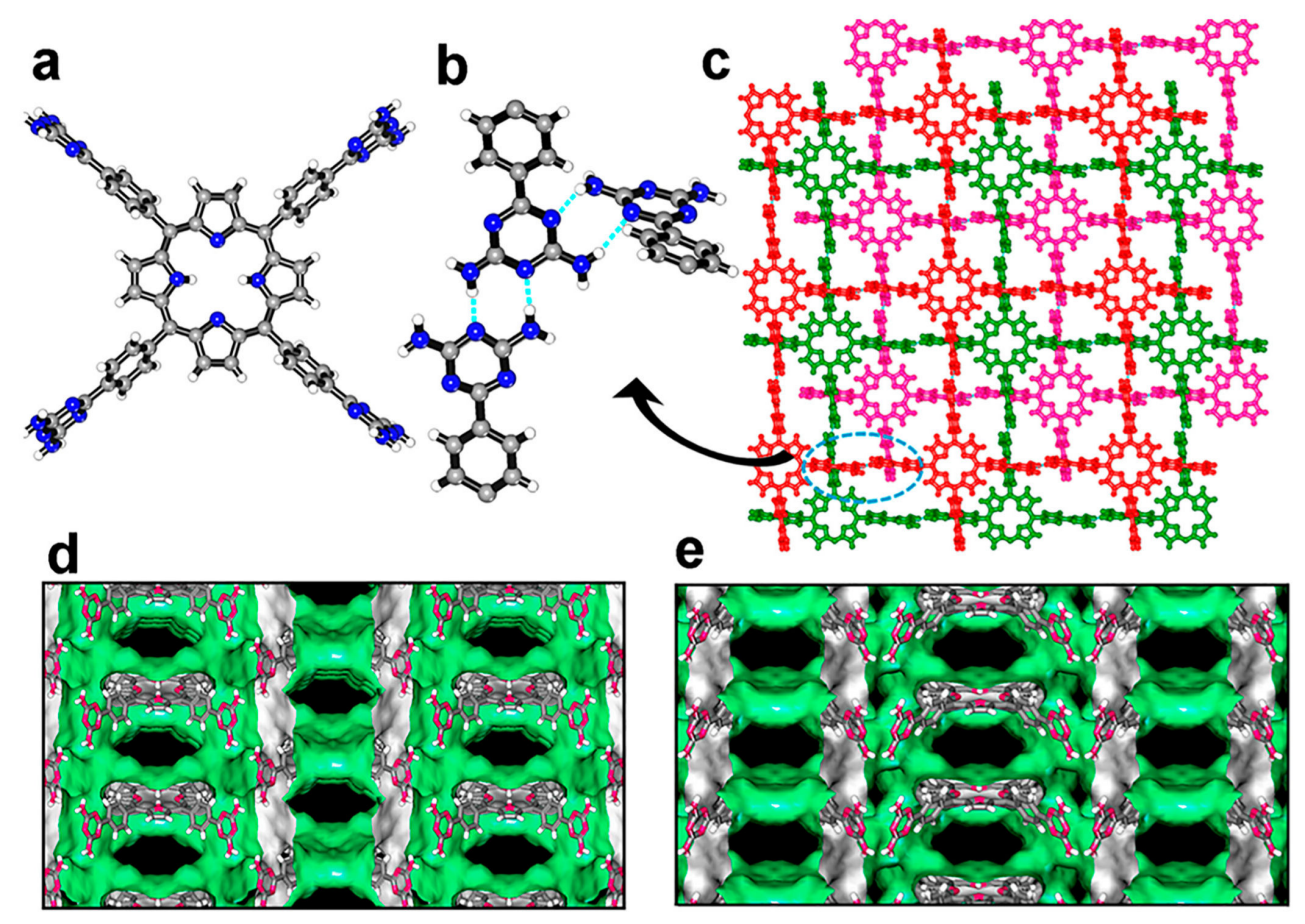
Schematic Molecular Structure of the HOF-6 Building Block; (**a**) building block of H_2_TDDP, (**b**) hydrogen-bonding interaction between DAT moieties, (**c**) three neighboring 2D supramolecular grids in different colors, (**d**,**e**) 3D packing supramolecular structure along the [1,0,1] and [1,0,0] directions with a channel size of ∼6.4 and 7.5 Å, respectively. Reprinted with permission from Ref. [49]. Copyright 2016 Copyright American Chemical Society.

In constructing amino-based HOFs, diamine units can form single-component HOFs with either water or other ligands like carboxyl or phosphate groups. For example, HOF-6, a metal-free porphyrin-based HOF, demonstrates permanent porosity and proton conductivity upon hydration (Figure 10) [49].

**Figure 11:**
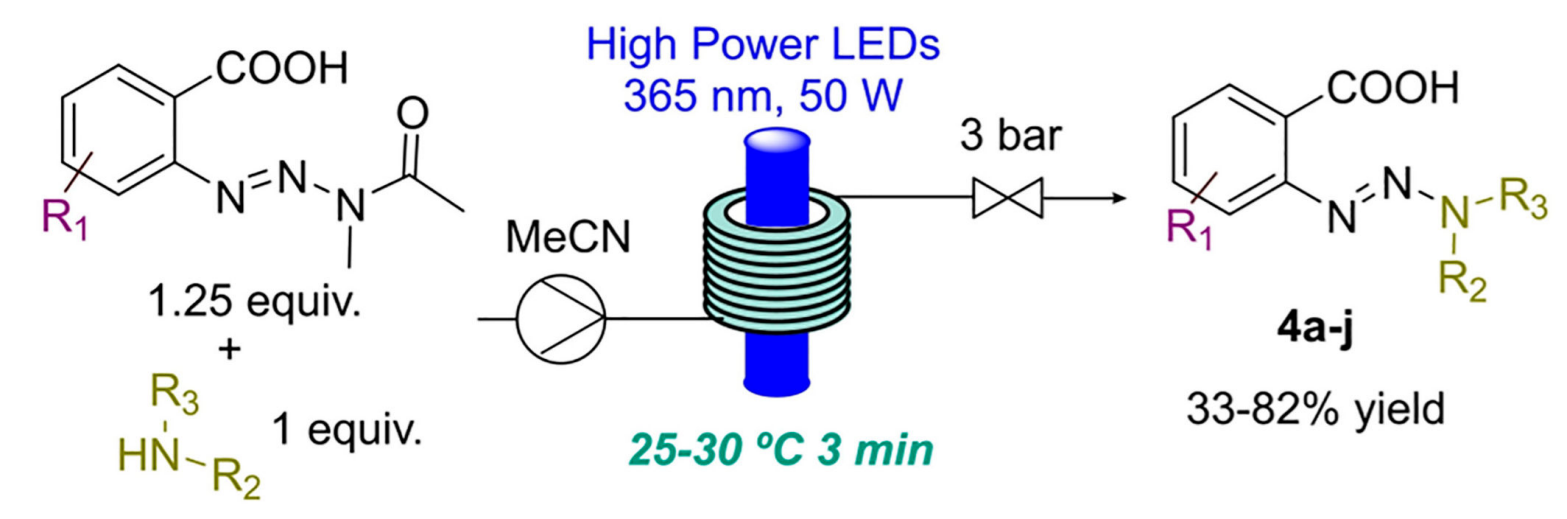
Scope of triazine formation in flow (Reprinted from Ref. [50]).

García-Lacuna, J. et al. presented a photochemical flow process using LEDs to synthesize phenylacetylene precursors. This metal- and photocatalyst-free method employs a modular approach, using a sequence of photo-flow transformations to produce benzotriazoles and aryl triazines in good yields, thus enhancing safety and scalability (Figure 11). This process employs a photocatalytic rearrangement reaction to generate a cyclic hydroxylamine intermediate through the photoexcitation of nitroaromatic compounds. The intermediate is then converted to phenylacetylene, which undergoes a second photochemical flow reaction to capture azides and benzotriazoles, producing heterocyclic target compounds. Notably, this modular technique also circumvents the necessity for hazardous, potentially explosive diazonium salts. The subsequent functionalization of the pendant carboxyl group results in the formation of biologically significant aryl triazine-based amides, thereby unlocking a myriad of applications for these distinctive and commercially valuable triazine compounds [50]. All cases indicated that continuous flow technology enables reactions to be carried out under safer conditions.

**Scheme 1:**
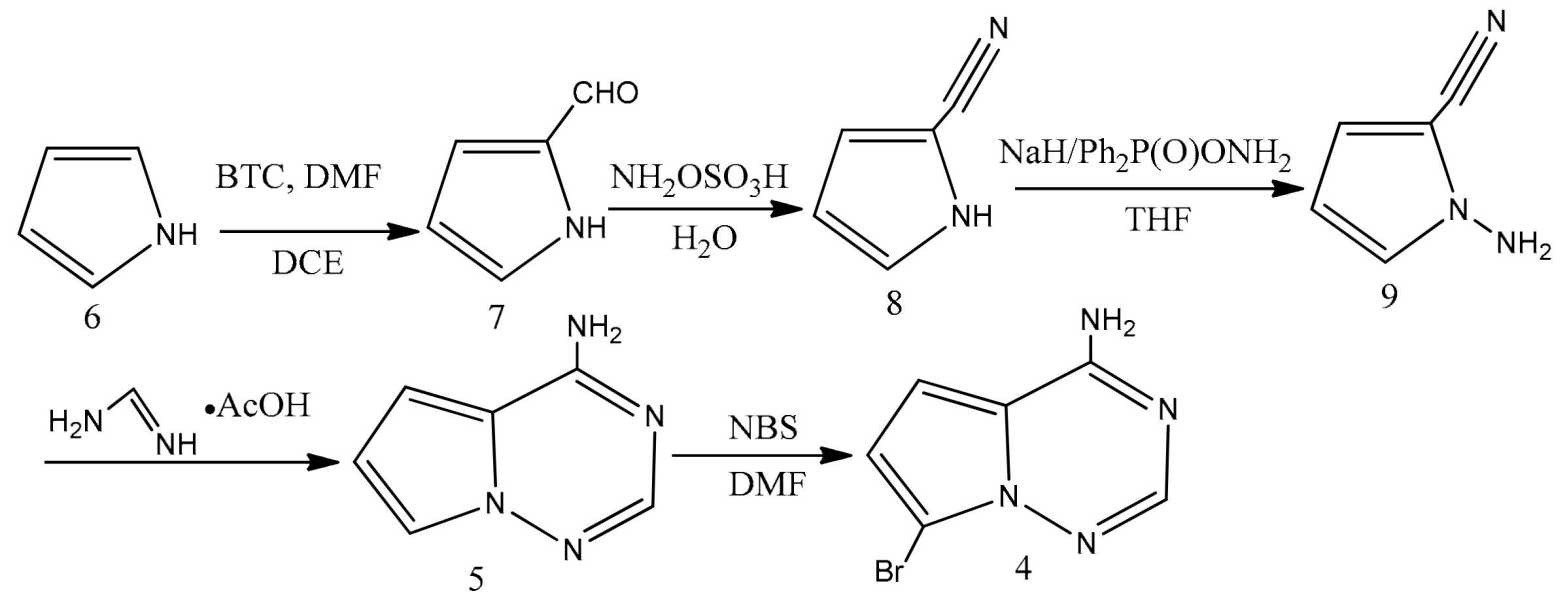
Improved synthetic protocol to prepare 7-bromopyrrolo [2,1-f] [1,2,4] triazin-4-amine (4) (Reprinted from Ref. [51]).

**Figure 12:**
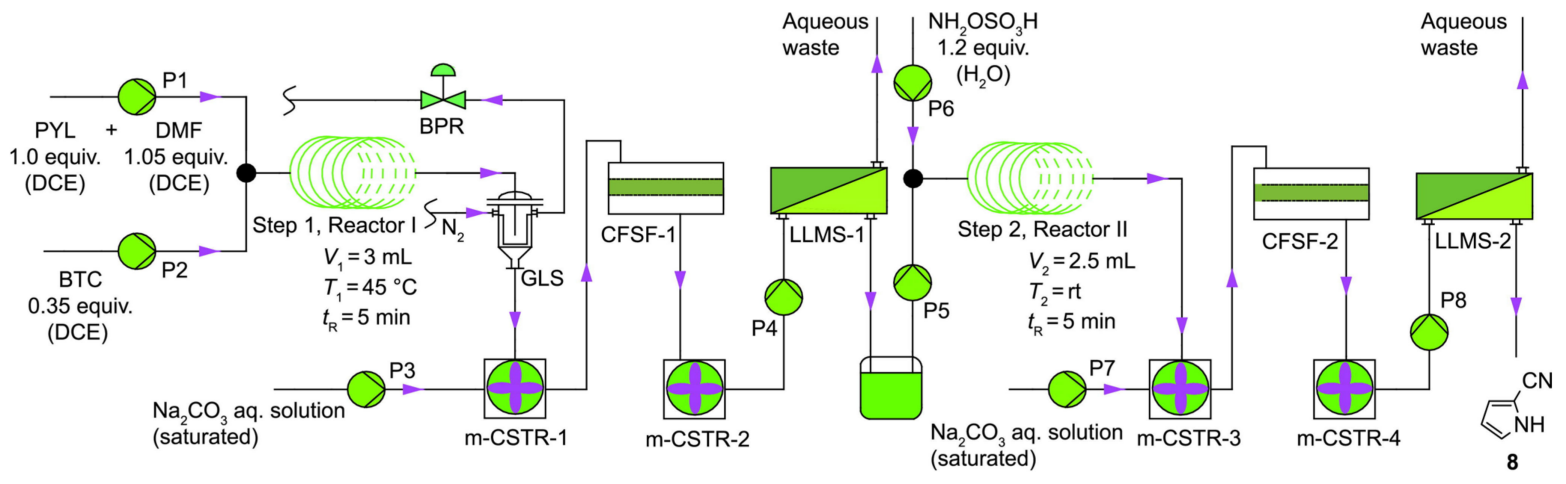
Schematic representation of the continuous-flow synthesis of 8. P20 and P21 are pumps (Reprinted from Ref. [51]).

The nucleobase unit of the remdesivir, 7-bromo-pyrrolo [2,1-f] [1,2,4] triazine-4-amine, was synthesized through multi-step continuous flow process using pyrrole as the raw material (Scheme 1). Under optimized flow conditions (reaction time of 79 min, yield of 14.1%), 7-bromo-pyrrolo [2,1-f] [1,2,4] triazine-4-amine was produced with a throughput of 2.96 g·h^−1^ (Figure 12). This continuous flow method significantly reduced the total reaction time compared to batch procedures (>26.5 h). Additionally, it facilitated highly exothermic reactions such as the Vilsmeier–Haack and N-amination reactions, which involve hazardous and unstable intermediates, as well as the bromination reaction requiring strict cryogenic control. The key advantage of this method lies in its integration of workup procedures directly within the reaction sequence, using specialized continuous flow devices. This integration maximizes process efficiency, achieving an eco-friendlier synthesis with improved safety and efficiency [51].

Differences among the above examples within the same functional group can include: substituent effects; steric effects; electronic effects; positional isomerism. The same synthesis method contains different process conditions, purification conditions, and synthesis scales.

### 2.4. Continuous Synthesis of Guanidines

Guanidine, a strong organic base (pKa = 13.6), forms a stable guanidinium ion upon protonation. In physiological environments, it typically exists in a fully protonated state, maintaining a positive charge across a wide pH range. This unique property enables guanidine to interact readily with ligands, receptors, and enzyme substrates through hydrogen bonding or electrostatic interactions, particularly due to its high affinity for phosphate esters, carboxylates, and metal ions [52,53,54].

**Figure 13:**
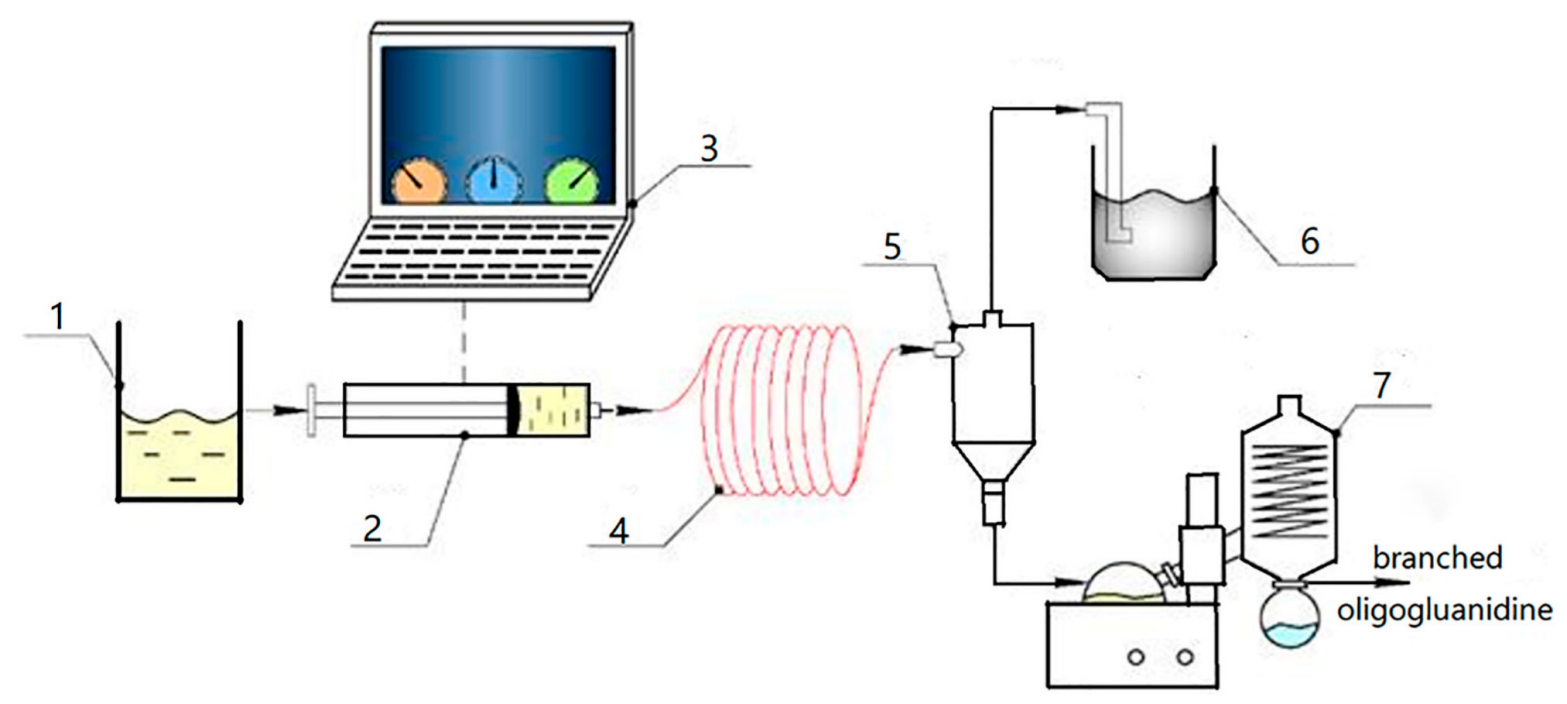
1-GH and HMDA aqueous solution; 2-medium-pressure syringe pump module; 3-PC; 4-microreactor; 5-cyclone; 6-flask-receiver of ammonia; 7-rotary evaporator (Reprinted from Ref. [55]).

Both polyguanidines and low-molecular-weight guanidine compounds exhibit superior antibacterial, antiviral, and antifungal activity compared to traditional antibiotics, without detectable bacterial resistance. This study used aqueous solutions of varying compositions to react guanidine hydrochloride (GH) with hexamethylenediamine (HMDA) under continuous-flow microfluidic conditions (Figure 13). The process produced a branched alkyl guanidine oligomer, at lower temperatures and with less time optimized for the desired oligomer properties. This work uses microfluidics technology to synthesize branched alkyl guanidine with potential antiviral activity, emphasizing the production of high-purity products with low residual monomer content (e.g., Mn = 800, branch number z = 0.4) [55].

**Figure 14:**
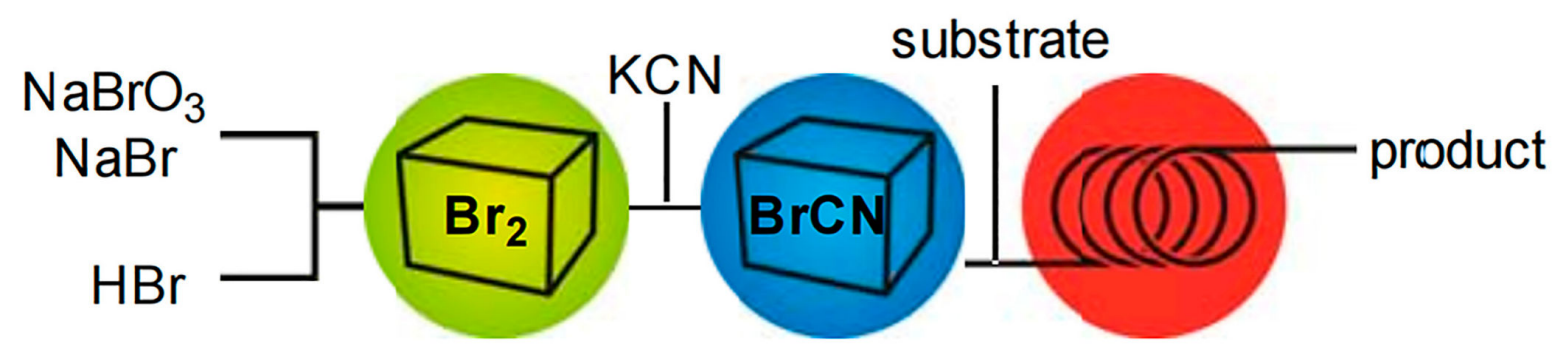
The BrCN generator set-up. PFA coil (0.8 mm i.d.) at 0–58 °C: 5.2 min residence time. Glass microreactor chip: 20 s residence time; liquid/liquid separator, FTIR. Reprinted with permission from Ref. [56]. Copyright 2017 Copyright John Wiley and Sons.

Through membrane separation technology, Glotz, G et.al designed a fully integrated continuous flow system, as shown in Figure 14. To reduce the handling and storage of elemental bromine, they added a continuous bromide generator based on the heterogeneous decomposition of bromate-bromide. Using online Fourier transform infrared spectroscopy (FTIR) technology, they can monitor and quantitatively analyze the generation of BrCN in real-time, with bromine (Br_2_) and the bromide generator producing 0.2 millimoles of BrCN per minute (with a concentration of 0.8 M in dichloromethane). This modular system is not only suitable for synthesizing various five- and six-membered cyclic amides and guanidines but can also operate in a fully integrated continuous mode or a semi-batch reaction crystallization mode [56]. Using continuous flow chemistry, bromine and BrCN are generated in situ for the synthesis of cyclic guanidine, which minimizes the handling and transportation of hazardous reagents in organic synthesis and pharmaceuticals.

### 2.5. Continuous Synthesis of Pyrazoles

Pyrazole plays a key role in the formation of HOF through hydrogen bonds. Donor-acceptor interactions are another reason for the π···π stacking between electron-rich pyrazoles. The versatile nature of substituents on the pyrazole ring has made it a core structure in various agricultural chemicals and pharmaceuticals. Continuous synthesis methods offer advantages in terms of on-demand production and efficient use of resources like time, space, and energy. 

**Figure 15:**
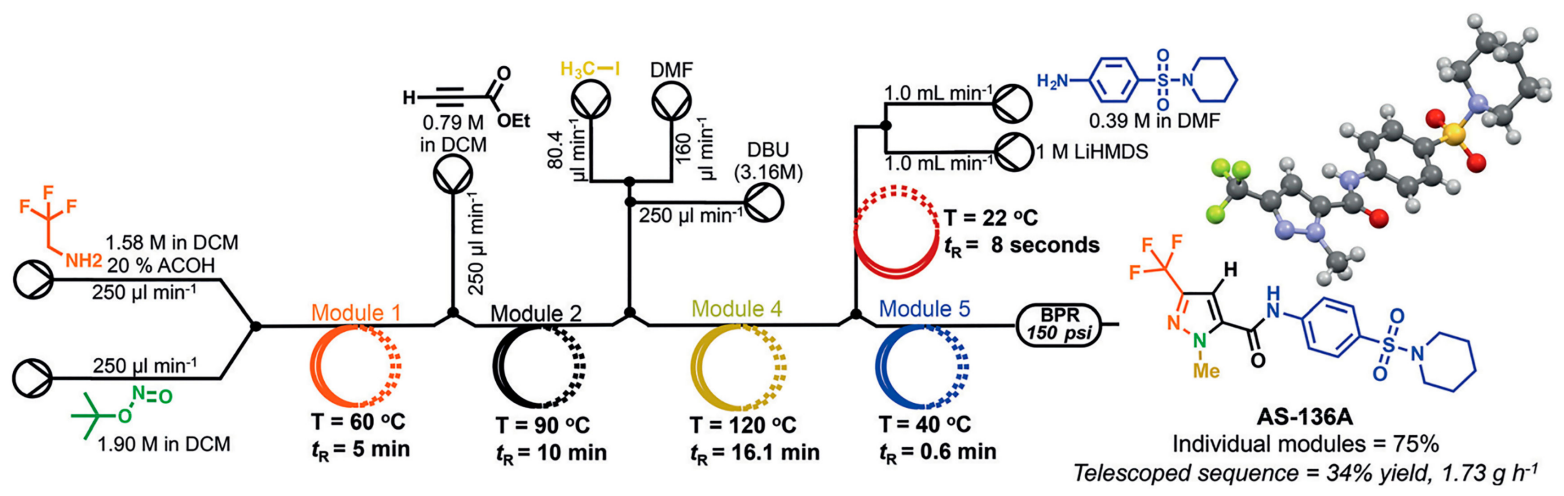
Continuous flow synthesis of AS-136A. A continuous flow telescoped synthesis with modules 1, 2, 4, and 5 to yield measles therapeutic AS-136A in a 34% isolated yield. Using modules individually yields AS-136A in a 75% yield. Reprinted with permission from Ref. [57]. Copyright 2017 Copyright John Wiley and Sons.

A continuous flow platform has been established for synthesizing valuable, functionalized pyrazoles and pyrazolines, producing a universal pyrazole core for subsequent modifications through a series of modules (Figure 15). By manipulating solvents at temperatures above the boiling points, the system enables rapid continuous flow reactions (1–60 min), enhancing reaction kinetics. This setup eliminates the need for quantitative Ag_2_O or bases as auxiliary reagents in [3+2] cycloaddition reactions with 2,2,2-trifluoromethyl diazomethane. Britton and Jamison utilized a continuous flow process to prepare fluorinated pyrazoles by generating CF_3_CHN_2_ in one tubular reactor and reacting it with alkynes in a second heated tubular reactor. This 3+2 cycloaddition is effective with electron-deficient aromatic alkynes, and notably, the reaction with CF_3_CHN_2_ proceeds without a metal catalyst [57,58].

**Figure 16:**
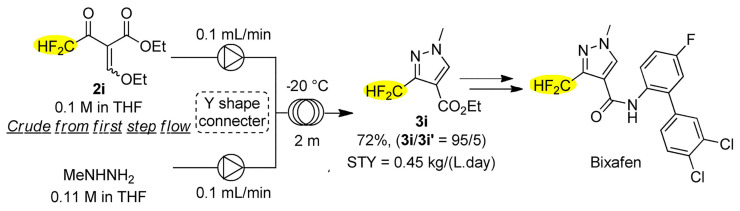
Continuous-flow synthesis of **3i** as a key intermediate. Reprinted with permission from Ref. [59]. Copyright 2019 Copyright John Wiley and Sons.

Das, A. et al. designed a two-step continuous flow method using vinyl ketate as a pivotal intermediate to synthesize substituted pyrazole derivatives (Figure 16). They found that Ni^2+^-montmorillonite is an effective catalyst for condensation of 1,3-dicarbonyl compounds, reducing the use of acetic anhydride and lowering the reaction temperature. These intermediates reacted with methyl hydrazine to produce pyrazole derivatives with high yield and selectivity under flow conditions. Moreover, Vinyl ketone esters are also shown as intermediates that can be converted into heterocyclic compounds, such as pyrimidine derivatives and the bioactive compound Bixafen [59].

The research is based on the current health challenges and drug needs to select continuous synthetic methods and functional groups, and the selection of these methods can not only improve the efficiency and safety of synthesis but also quickly respond to the changes in the medical and agricultural fields.

### 2.6. Continuous Synthesis Imidazole

Imidazole is a planar, five-membered heterocycle with the chemical formula C_3_H_4_N_2_, containing two nitrogen atoms, three carbon atoms, and four hydrogen atoms. Its aromaticity is due to a 6-electron conjugated large π bond in the molecules, the nitrogen atom bonded to hydrogen with a σ bond provides a pair of electrons, and the other four atoms in the ring each provide one electron for bonding [60]. It can form many forms of hydrogen bonds. The diffusion of gas-phase imidazole into the pores of HOFs further improves its proton conductivity [61].

**Figure 17:**
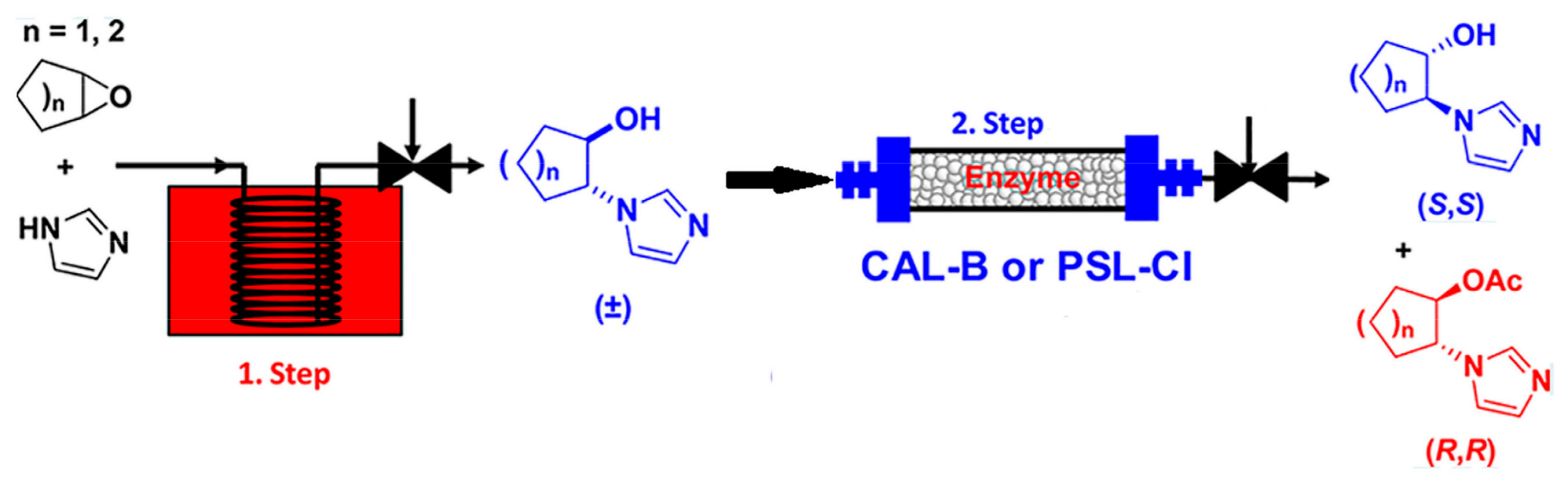
Schematic setup for the epoxide ring-opening reaction under flow conditions. Enzymatic kinetic Resolution of Racemic trans-2-(1H-imidazol-1-yl) cycloalkanols trans-3a-b in fixed bed. Reprinted with permission from Ref. [62]. Copyright 2012 Copyright American Chemical Society.

Advancements in continuous-flow synthesis have enabled efficient production of chiral, enantiopure 1-(2-hydroxycycloalkyl) imidazoles, achieving up to 99% conversion with a productivity of 9.9 g·h^−1^ within a 12 min residence time. Furthermore, continuous-flow systems have proven superior for the lipase-catalyzed kinetic resolution of racemic 2-(1H-imidazole-yl) cycloalkanes, utilizing immobilized CAL-B or PSL-C enzymes, and enabling scalable production of chiral imidazoles, which are essential in synthesizing chiral ionic liquids (Figure 17) [62].

**Scheme 2:**
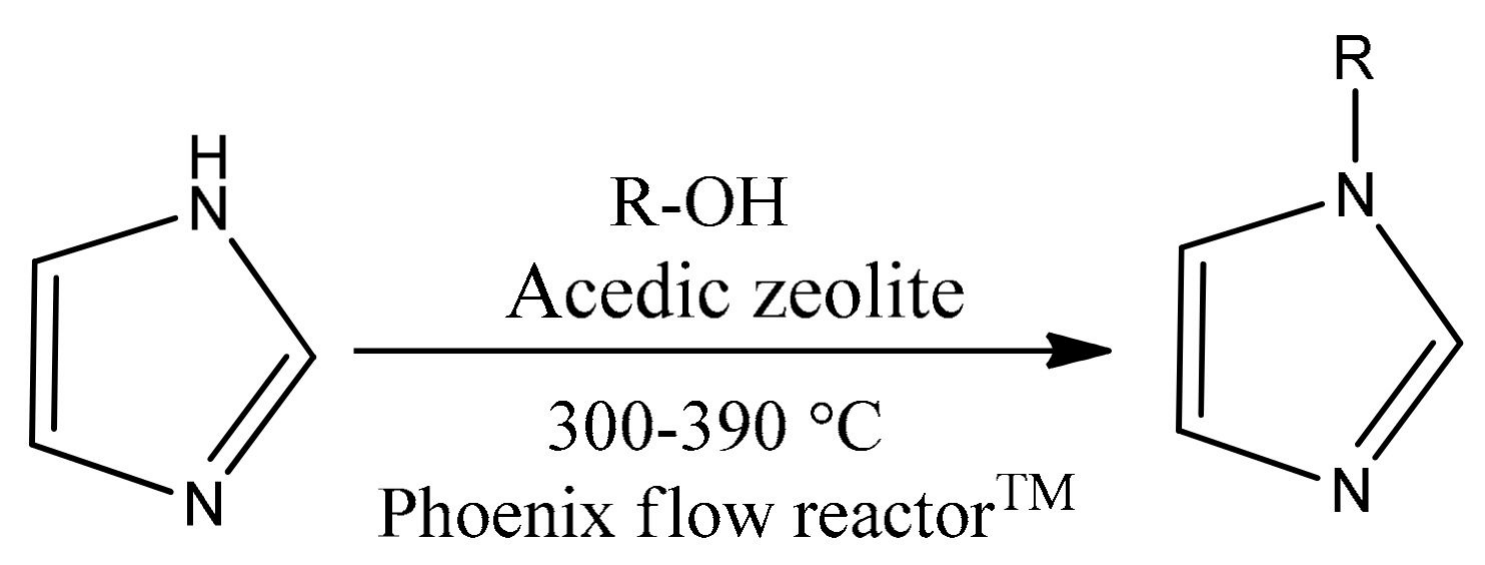
One-step continuous flow synthesis of *N*-alkyl imidazoles (Reprinted from Ref. [63]).

In a fixed-bed reactor, Melinda Fekete et al. demonstrated the continuous-flow synthesis of *N*-alkyl imidazoles, which are key components in ionic liquids. Using acidic molecular sieve catalysts under high temperatures (>300 °C) and high pressure conditions, the team achieved yields of 9–14 g/h and selectivity over 95%, with the catalyst remaining active for over 20 h (Scheme 2) [63].

**Figure 18:**
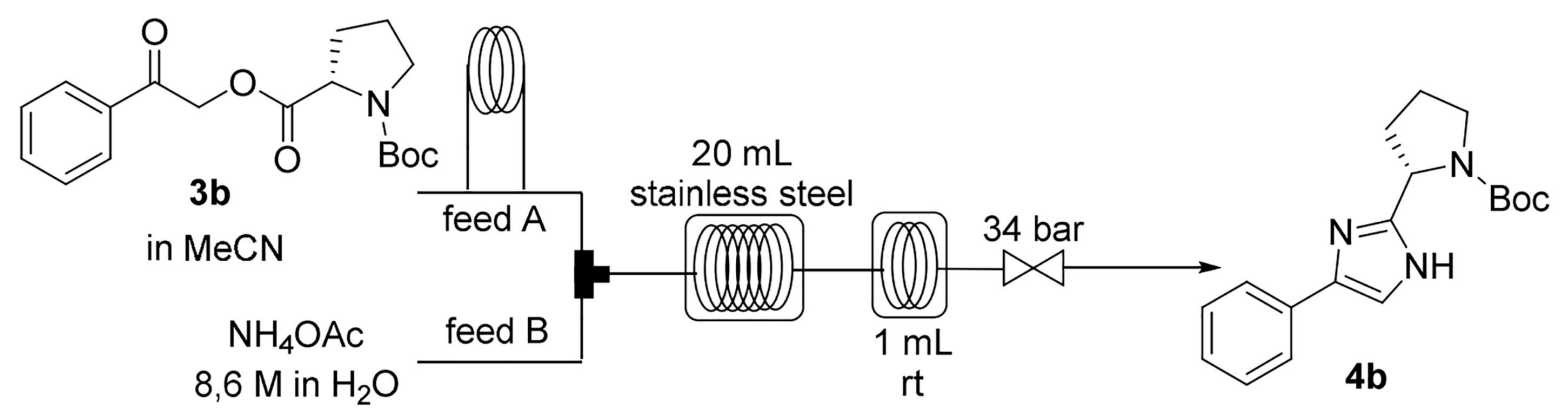
Continuous Flow Formation of Imidazole 4b from Acyloxy Ketone **3b**. Reprinted with permission from Ref. [64]. Copyright 2015 Copyright American Chemical Society.

In another example, alpha acyloxyketone was first synthesized from alpha bromoacetophenone and carboxylic acid, followed by condensation with ammonium acetate in a stainless-steel coil reactor (150 °C, 17 bar) producing high-purity 1H-4-substituted imidazoles. The continuous flow system facilitated rapid heating of the regent’s mixture to the target temperature and pressure, and increased the concentration of volatile compounds, allowing for imidazole formation within a 2 to 5 min residence time (Figure 18) [64].

**Figure 19:**
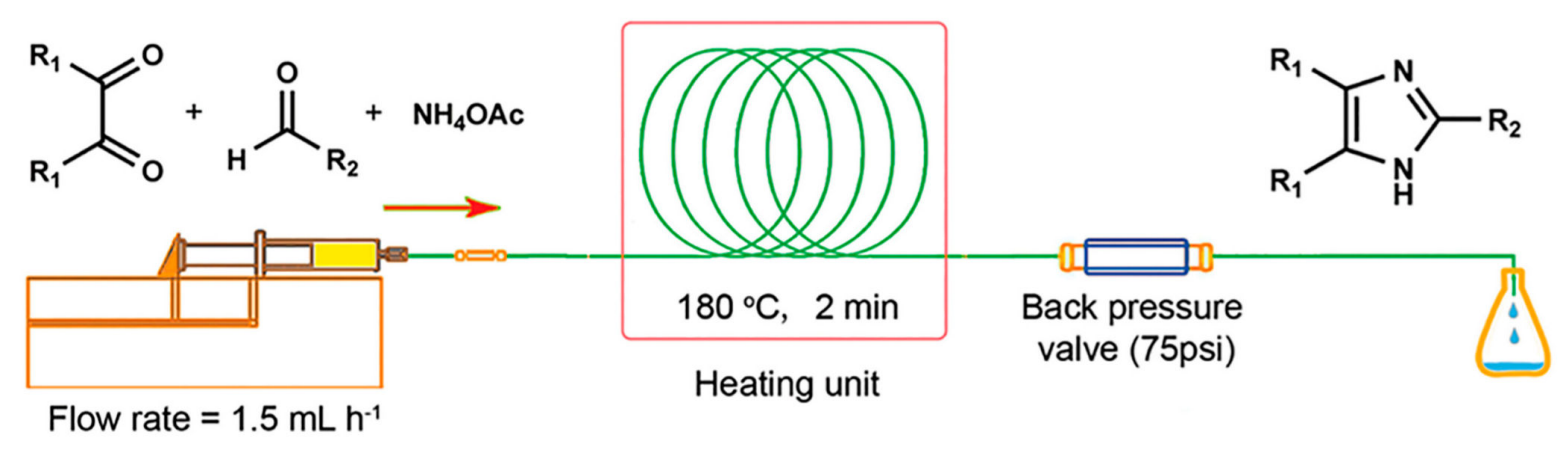
Continuous synthesis of 2,4,5-trisubstituted imidazoles using a microreactor system under pressure. Reprinted with permission from Ref. [65]. Copyright 2010 Copyright American Chemical Society.

A new universal microreactor method has also been developed for synthesizing 2,4,5-trisubstituted imidazoles. The system includes a microinjection pump, microchannels, a heating unit, a back-pressure (75 psi) valve, and a Cone-shaped bottle. Various substrates, including aromatic and aliphatic aldehydes are processed within 2 min at 180 °C in the 120-cm-long stainless-steel tube. The microreactor produced high yields of aryl, alkyl, and heteroaromatic imidazoles under superheated conditions (Figure 19) [65].

**Scheme 3:**
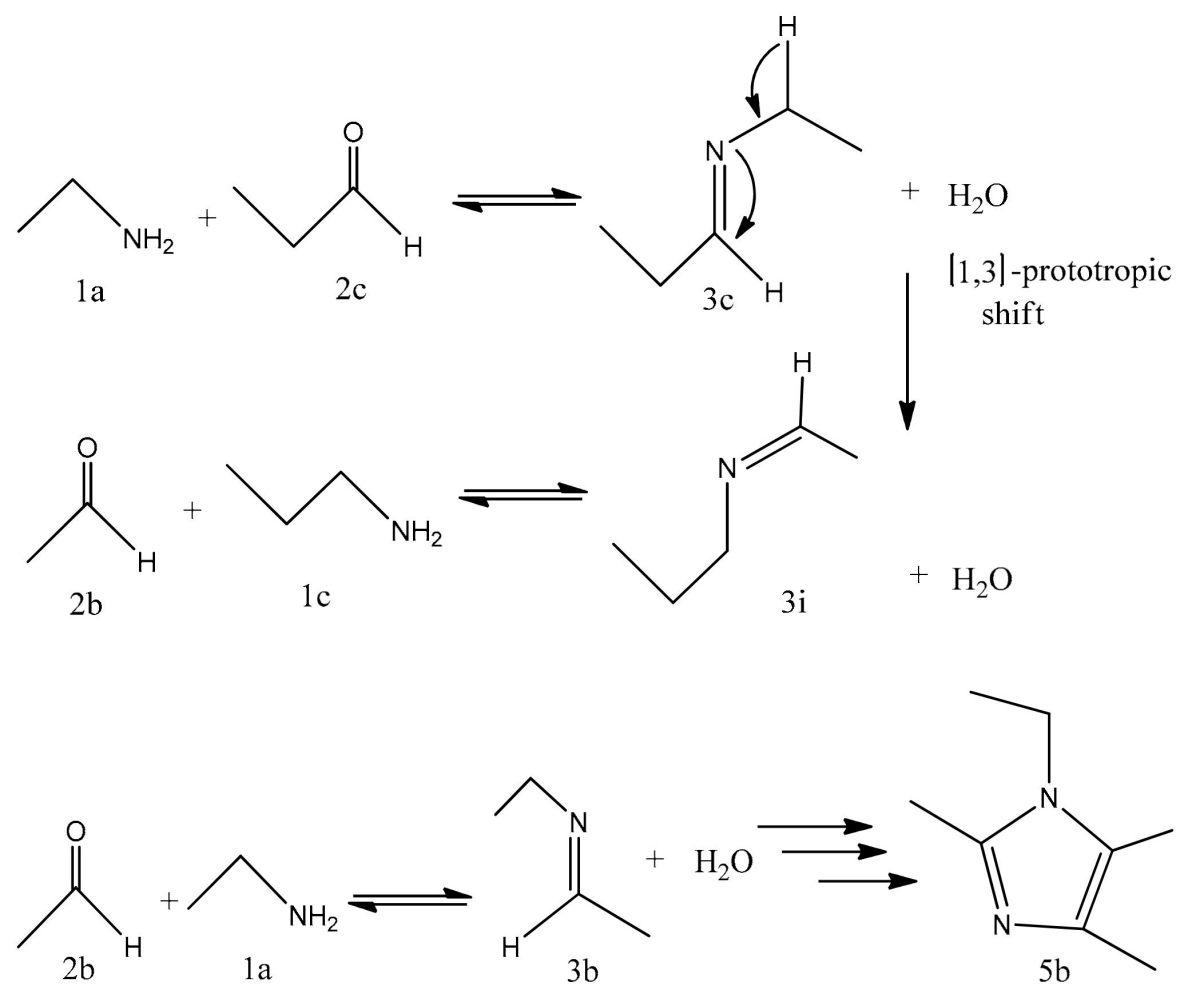
[1.3]-prototropic shift of ethylpropylideneamine and consecutive formation of 1-ethyl-2, 4, 5-trimethylimidazole in alkaline media [48].

Polyalkylated imidazoles with strong chemical stability under alkaline conditions have also been synthesized via continuous-flow chemistry. These imidazoles are then converted into ionic liquids. Compared to traditional imidazole-based ionic liquids, those produced via continuous flow offer enhanced thermal and electrochemical stability (Scheme 3) [48].

Above all, the continuous flow process has the advantages of high heat and mass transfer, ease of scaling, and possible improvement of safety due to the closed nature of the process; due to its characteristics of numbering-up, it provides a scalable solution; imidazole derivatives can achieve high-temperature continuous and stable synthesis in different microreactors.

### 2.7. Continuous Synthesis of Pyridine

Pyridine and its condensed derivatives, such as pyrimidine, are critical components of nucleic acids and vitamins, playing essential roles in various treatments. This unique heterocyclic system naturally complements biological structures, allowing for the development of new derivatives with important biological and medical applications [66]. The interactions between different functional groups contained in pyridine-2,6-dicarboxylic acid can self-assemble into HOF materials [67].

A microwave flow reactor has been developed for the one-step synthesis of pyridines and dihydropyridines via the Bohlmann–Rahtz or Hantzsch multicomponent reactions, respectively. The Bohlmann–Rahtz reaction uses a Brønsted acid to catalyze Michael addition and cyclodehydration, yielding trisubstituted pyridine in a single regioisomer. This predictable process is well-suited for scaling up, with continuous-flow microwave processing achieving high yields [20]. 

In a two-step approach, 4-nitropyridine was successfully synthesized from pyridine *N*-oxide. Nitration with HNO_3_ and H_2_SO_4_ produced 4-nitropyridine N-oxide, which was further reacted with PCl_3_ to yield the final product. Continuous flow minimized the accumulation of energetic nitration intermediates, enhancing safety and scalability. Continuous extraction during nitration enabled a daily throughput of 0.716 kg of 4-nitropyridine with 83% yield and high selectivity [68].

To address coking deposits in commercial pyridine synthesis reactors, a coupled fluidized bed reactor was developed, comprising a feeding zone (FZ), riser reaction zone (RRZ), and fluidized bed reaction zone (FRZ). After running continuously for 15 days, no coke deposit was formed. Experimental results showed product yields as high as 75%, with selectivity outperforming conventional reactors. Modeling results confirmed that the main reactions occur in the FZ and FRZ, while RRZ contributed minimally due to mass transfer limitations [69].

**Scheme 4:**
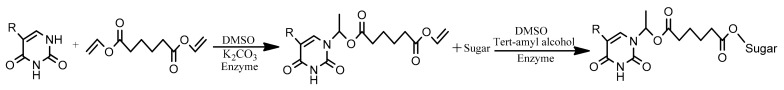
Synthesis of sugar-containing pyrimidine compounds in continuous-flow microreactors (Reprinted from Ref. [70]).

The two-step continuous flow Scheme 4 can effectively and gently obtain the target compound (step 1 at 40 °C, step 2 at 30 °C). By extending the Markovnikov addition and esterification steps into a single, continuous, and uninterrupted reactor network, the need for separation and purification of intermediate products is avoided. The scope of the reaction is tested by changing the pyrimidine derivatives, vinyl esters, and sugars. The method reduces the use of DMSO, shortens the reaction time (step 1 10 min, step 2 30 min), and uses enzymes as catalysts to achieve high regional selectivity. They can use this technology to quickly synthesize a related compound library in preparation for the next step of drug activity screening [70].

**Figure 20:**
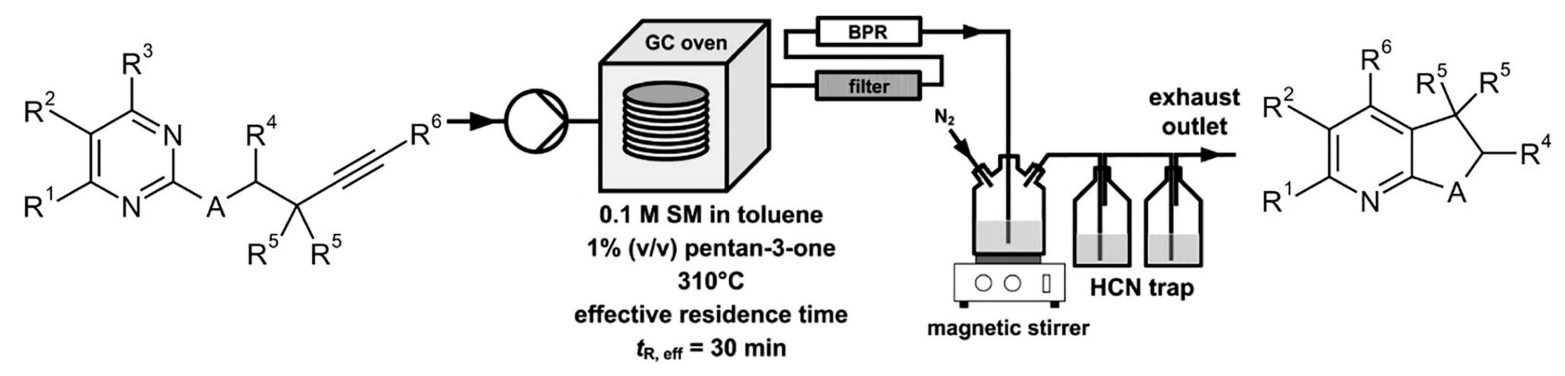
Synthesis of annulated pyridines in flow. Reprinted with permission from Ref. [71]. Copyright 2012 Copyright John Wiley and Sons.

Martin, R. E and colleagues demonstrated that pyrimidine alkynes could be transformed into annulated pyridines via a cascade of HDA reactions and cycloreversions, achieving high yields. Flow technology allowed solvents to be superheated, thus avoiding hazardous solvents like nitrobenzene. This process also safely trapped cyano side products continuously, preventing accumulation and facilitating scalability. The addition of pentane-3-one (1% v/v) to the toluene solvent stream was crucial to prevent blockage and ensure scalability (Figure 20) [71].

Substrates with electron-withdrawing groups attached to atom A (such as fluorine, chlorine, bromine, or nitro) typically exhibit higher reactivity, resulting in higher yields of cyclized pyridine products. Switching the connecting atom A from carbon to oxygen and nitrogen resulted in a significant decrease in yield.

### 2.8. Continuous Synthesis of Thiazole 

Thiazole is a five-membered aromatic ring containing sulfur and nitrogen atoms. Due to its electron-rich heteroatoms, thiazole can form coordination bonds with transition metals and hydrogen bonds with nitrogen, oxygen, and fluorine atoms. Thiazoles are typically synthesized by the condensation of thioamides with α-bromoketones using the Hantzsch method [72,73,74]. Their derivatives have special anti-solvent color effects through the formation of hydrogen bond networks and were applied in the rapid detection of trace water [75].

**Figure 21:**
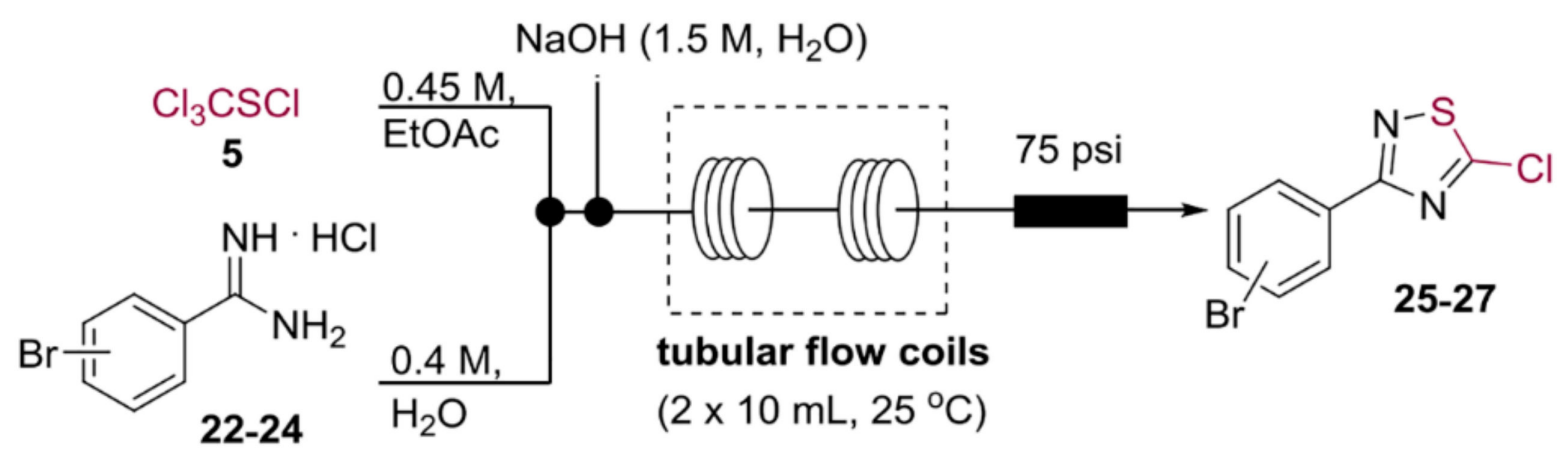
Flow synthesis of thiadiazoles. Reprinted with permission from Ref. [76]. Copyright 2017 Copyright Elsevier.

A continuous-flow process has been developed for synthesizing and derivatizing 1,2,4-thiadiazole heterocycles (Figure 21). Special attention was given to safely handling trichloromethane sulfenyl chloride by in-line quenching, eliminating malodorous and corrosive by-products. This process enabled the safe synthesis of gram quantities of 5-chloro-3-phenyl-1,2,4-thiadiazole, which was subsequently diversified using nitrogen-, sulfur-, and oxygen-based nucleophiles [76].

A straightforward protocol was also developed for synthesizing imidazo [2,1-b] thiazoles, starting from benzaldehydes, 2-aminothiazoles, and alkynes under copper catalysis. The reaction produced a variety of aryl-substituted imidazo [2,1-b] thiazoles and thiazoles with yields ranging from 33% to 93%. Continuous-flow intensification further increased yields up to quantitative levels [77].

**Figure 22:**
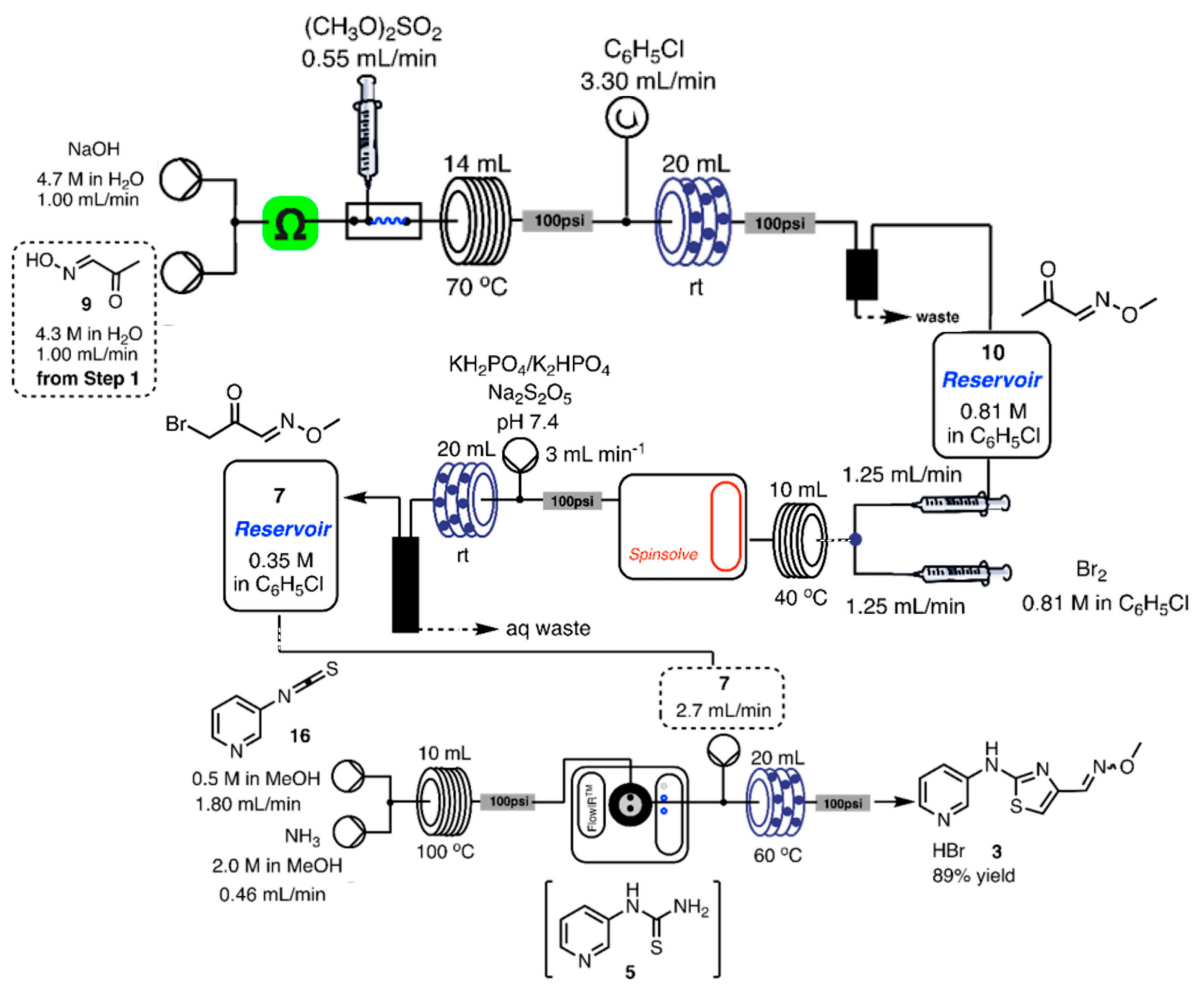
Fully telescoped synthesis of the thiazole target **3** (Reprinted from Ref. [78]).

Godineau, E. et al. designed a flow process for assembling C4-oxime-substituted thiazoles, using 3-bromo-2-oxopropanal O-methyl oxime as a key intermediate (Figure 22). This three-step sequence increased robustness and on-demand generation efficiency, improving scalability and safety. The continuous-flow setup enabled the simultaneous synthesis of unstable intermediates for convergent synthesis, with real-time NMR and IR analysis integrated into the process [78].

Each method serves its purpose in advancing synthetic chemistry, with a specific emphasis on improving yield, safety, or substrate diversity. The papers do not provide sufficient mechanistic insight into how the flow process and in-line quenching work on a molecular level, which would be valuable for readers seeking a deeper understanding.

### 2.9. Continuous Synthesis of Triazole, Tetrazole

Triazole is a five-membered aromatic heterocycle, conducive to forming various non-covalent bonds, making it effective in promoting the binding of proteins, enzymes, and receptors and demonstrating extensive biological and pharmacological activities. Tetrazole, containing four nitrogen atoms, has a planar ring structure and an electron-rich conjugated system, allowing it to participate in various non-covalent interactions. The synthesis of multicyclic functionally substituted triazole- and tetrazole-containing products has increased the possibility of obtaining various HOF materials [79]. The attachment of triazole derivative to the pores of the MOF or the combination of HOF and MOF greatly improves its proton conductivity [80].

#### 2.9.1. Continuous Synthesis of Triazole

Francesco Ferlin reported an efficient method for C-H functionalization of 1,2,3-triazoles under continuous flow conditions, applicable to various functional groups and either inter- or intramolecular reactions. This continuous flow system uses heterogeneous Pd/C catalysts, soluble organic bases, and γ-valerolactone (GVL) as the reaction medium. Under optimized conditions, the combination of tetrabutylammonium acetate (TBAAc) and tetrabutylammonium iodide (TBAI) achieved a conversion rate of up to 97% and a separation yield of 87%. The system enables efficient recovery and extended stability of the catalysts [81]. In continuous microreactor system, organic azides are prepared in situ and subjected to [3 + 2] cycloaddition reactions with cyanoacetamide, generating various substituted 1,2,3-triazoles, which are further functionalized to create a core structure like that of the antiplatelet drug Brilinta^®^. This method is well-suited for flow chemistry as it maintains continuous heating and mixing while eliminating the need to isolate intermediate azides [82]. The references [81,82] focus on the functionalization of 1,2,3-triazoles, with the [81] using a palladium catalyst for C-H activation and the [82] emphasizing the safety improvement through the in-situ generation of azides.

**Figure 23:**
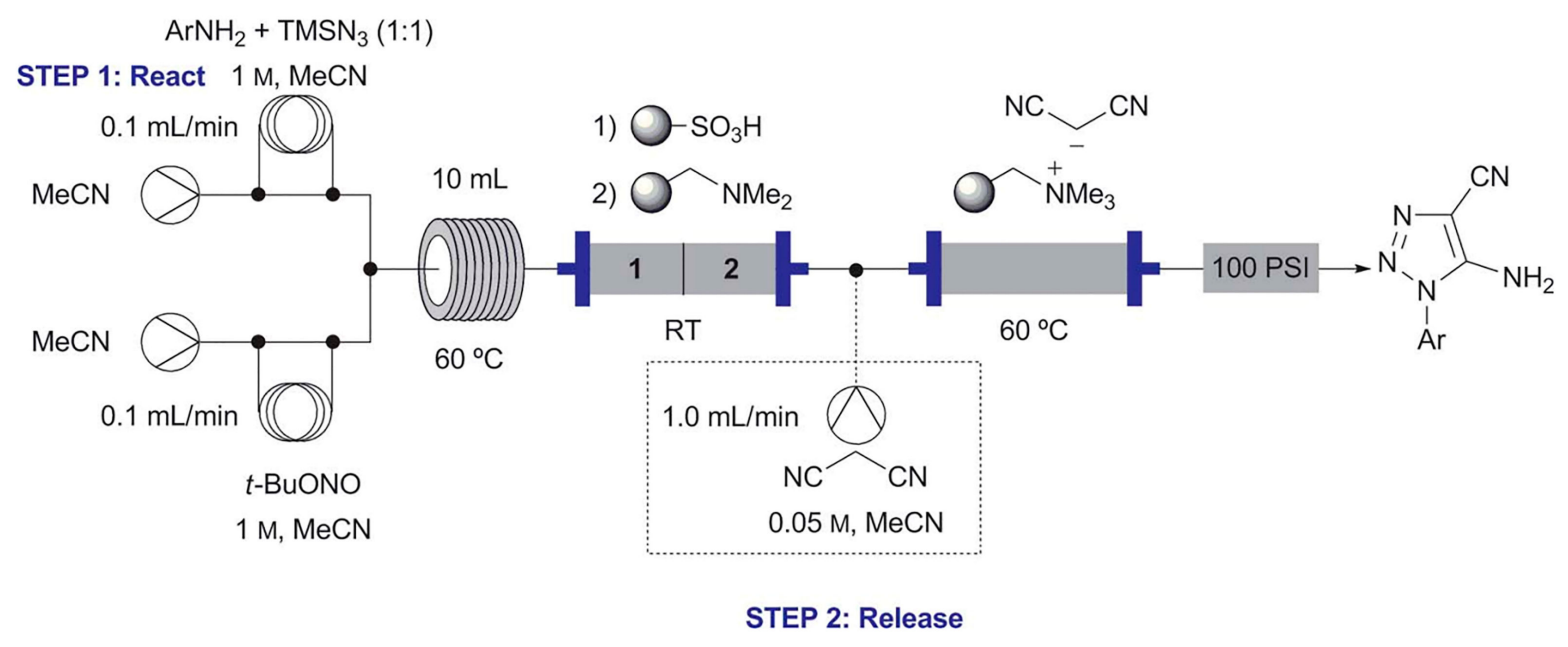
Flow synthesis of 5-amino-4-cyano-1,2,3-triazole. Reprinted with permission from Ref. [83]. Copyright 2024 Copyright American Chemical Society.

Catherine J. Smith et al. described an online purification process using polymer-bound sulfonic acid (QP-SA) and dimethylamine (QP-DMA) to improve reaction safety in synthesizing 5-amino-4-cyanomethyl-1,2,3-triazole from aniline starting materials. This automated continuous flow process demonstrates the potential for long-duration, fully automated chemical process flows, achieving up to 55 h of synthesis and showcasing the capabilities of process automation in chemical synthesis (Figure 23) [83].

**Figure 24:**
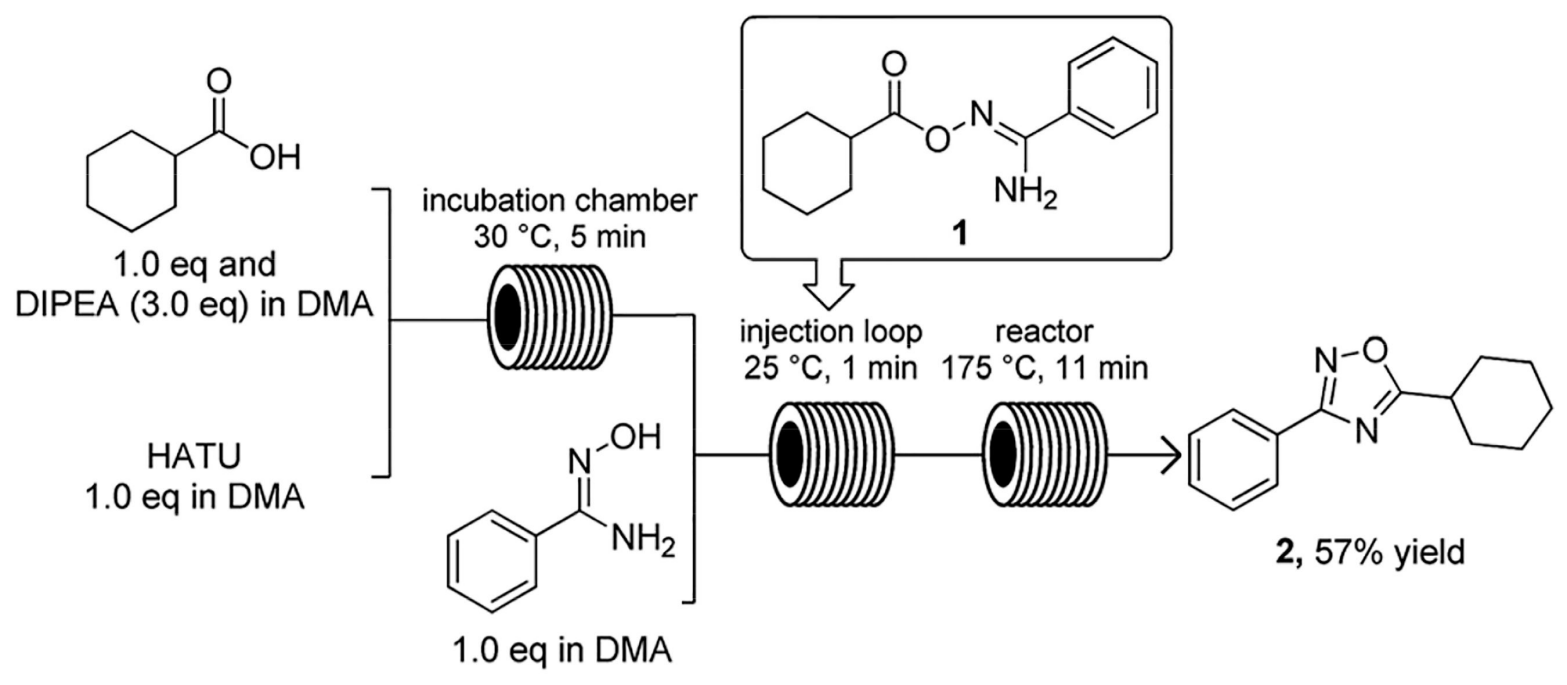
Continuous flow synthesis of 1,2,4-oxadiazole 2 (Reprinted from Ref. [84]).

Researchers have developed a high-throughput method for synthesizing a small molecule library of 1,2,4-oxadiazoles and 1,2,4-triazoles using a continuous flow reactor within an integrated synthetic purification platform (Figure 24). First, various carboxylic acids react with hydroxylamine, hydrazone amine, or pyridyl hydrazine through low-temperature peptide coupling, forming intermediates. These intermediates then undergo high-temperature cyclization to yield the desired heterocyclic compounds. This platform enables rapid library generation and accelerates the drug discovery process. The authors also demonstrated the SWIFT (Synthesis with Integrated Flow Technology) system, utilizing the Accendo Conjure flow reactor, which uses segmented flow with immiscible fluorine-containing spacers to create distinct reaction segments. With optimized cyclization temperatures (150–175 °C), this approach achieved high yields for various 1,2,4-oxadiazoles and 1,2,4-triazoles. The system demonstrated that compounds synthesized from hydrazine and 2-hydrazine pyridine can react with a range of aromatic and aliphatic carboxylic acid monomers, obtaining target compounds with moderate to high yields [84]. The results show the application of continuous flow chemistry in drug discovery. Although these examples emphasize efficiency and sustainability, they also demonstrate rapid synthesis and purification for screening.

**Figure 25:**
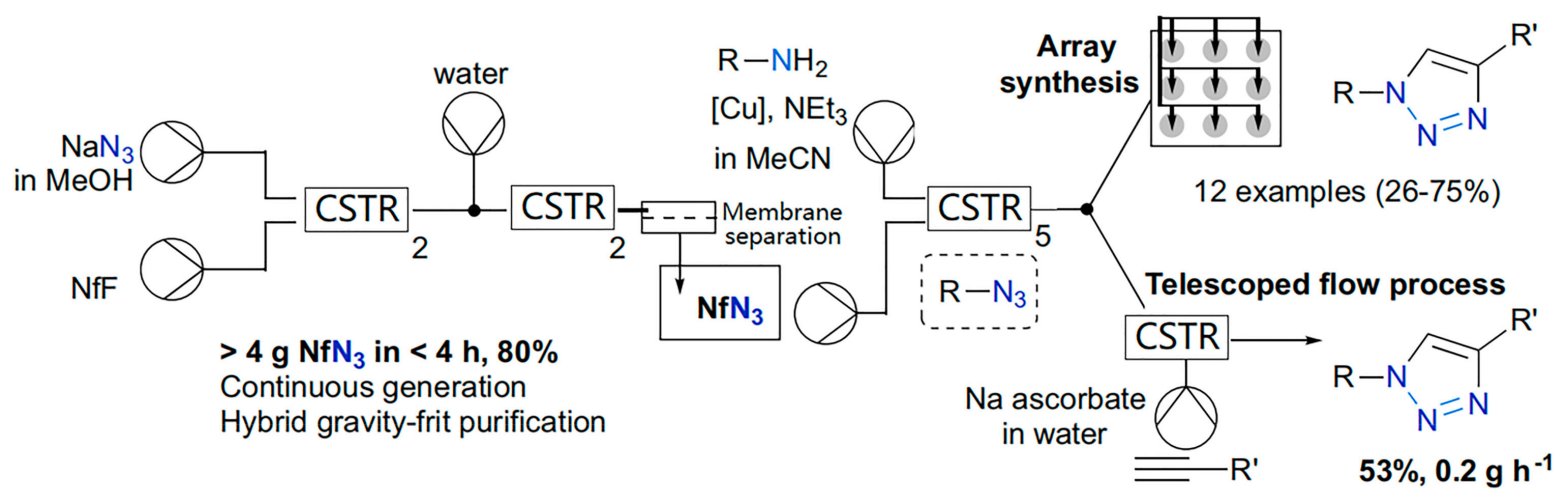
This work is to develop a three-step flow preparation of nonafyl azide, diazo transfer to primary amines, and subsequent CuAAC formation of triazoles (Reprinted from Ref. [86]).

Efficient synthesis of phenylglucososazone was achieved in a continuous flow process by reacting glucose with phenylhydrazine under acidic conditions. The process was further optimized to convert phenylglucososazone into 2-phenyl-1,2,3-triazole through oxidative cyclization. Reflux heating with copper (II) sulfate as an oxidant ensured high triazole yields, while continuous flow operation prevented reactor blockages. Using NaIO_4_ for diol cleavage, valuable triazole carboxaldehyde building blocks were also obtained [85]. The diazo transfer reagent NfN_3_ was successfully synthesized through a two-step continuous flow chemistry method, enabling the preparation of multiple triazoles, including the antiepileptic drug Rufinamide, from primary amines and alkynes via a semi-batch CuAAC click reaction (Figure 25). This reaction sequence bypasses the need to isolate alkyl azide intermediates and can also synthesize organic azides [86]. The first example involves reactor fouling and scalability issues, while the second example focuses on improving the safety and efficiency of treating reactive azide compounds. The selection of functional groups—benzyl glucosamine azide and azide/triazole—reflects different synthetic goals, with the former emphasizing the generation of heterocyclic skeletons and the latter focusing on the transformation of intermediate azide compounds. Although both methods show potential, there is still room for further optimization in terms of improving yield and sustainability.

**Figure 26:**
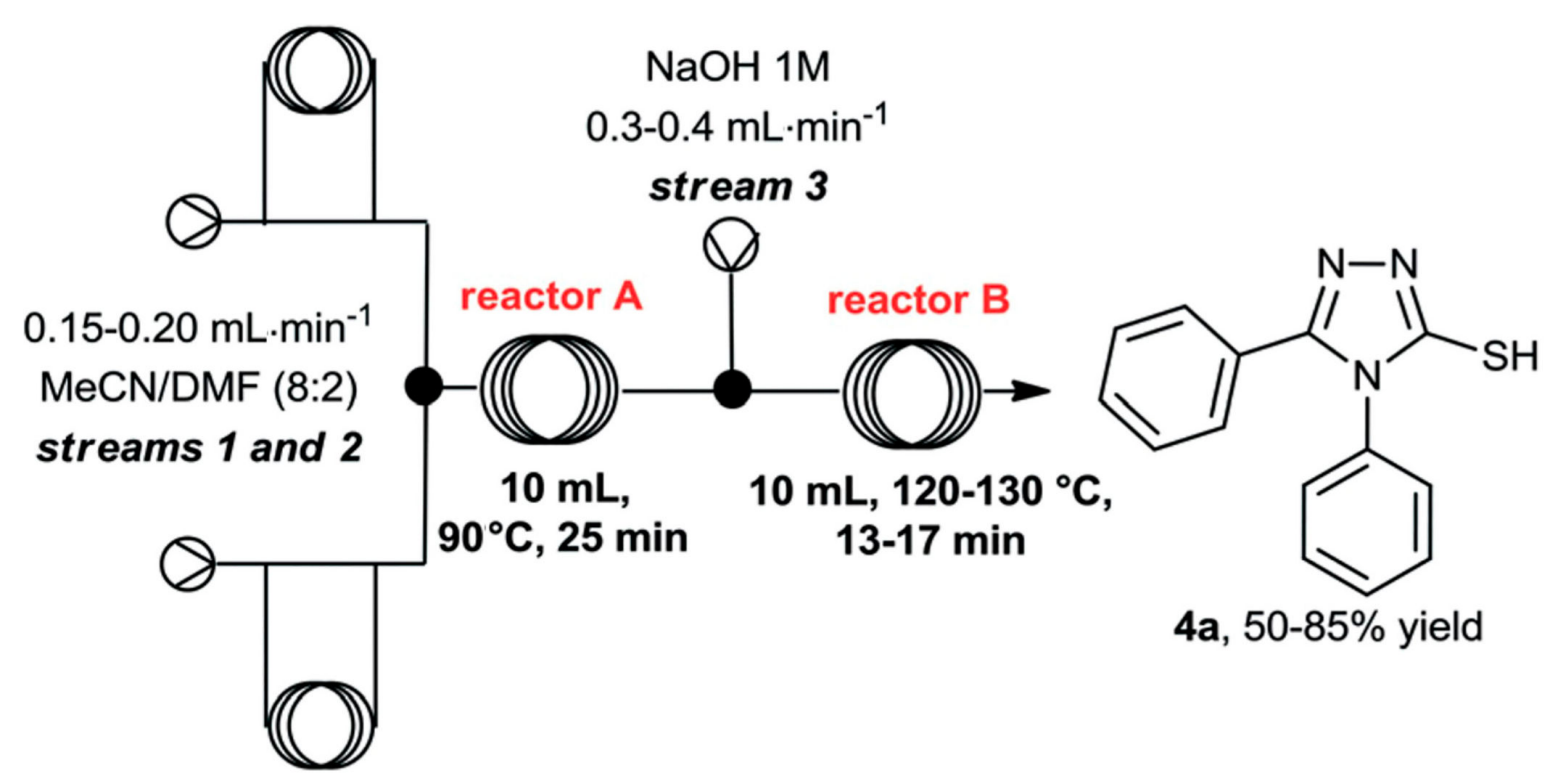
Evaluation of the conditions for step A and B-cyclization in continuous flow regime [88].

Starting from 2,4,6-trimethylaniline, diazotization yields a diazonium salt that couples with 1H-1,2,4-triazole-3-thiol before undergoing diazotization to produce 3-(mesitylthio)-1H-1,2,4-triazole. Continuous flow technology effectively suppresses side reactions such as hydrolysis, dimerization, and intramolecular coupling of diazonium salts, resulting in a yield increase from 60% to 85% by optimizing reaction conditions [87]. Damiao, M. et al. developed a fully integrated continuous flow process to efficiently synthesize highly substituted 3-thiophene-1,2,4-triazoles (Figure 26). Through a condensation reaction of hydrazine and isothiocyanate (step A), an in-situ stream of thiocysteamine was generated and cyclized under alkaline conditions (step B), yielding 1,2,4-triazole-3-thiol, which was further alkylated with benzyl/alkyl halides. This platform synthesized a library of 18 compounds with a residence time of 48 min per synthesis, achieving moderate to excellent yields [88]. The use of continuous flow technology to synthesize the target product exhibits fast and efficient characteristics. Both examples emphasize the advantages of continuous flow chemistry in improving reaction efficiency, reducing side reactions, and achieving large-scale or high-throughput synthesis. Ref. [87] focuses on a specific reaction to produce 3-methylthio)-1H-1,2,4-triazole with high yield and purity, while [88] uses continuous flow chemistry to quickly generate a series of functionalized 3-thio-1,2,4-diazacyclic compounds for drug discovery. The second example demonstrates more flexibility in generating various compounds, making it particularly useful in early drug development. However, both methods can be further optimized in terms of environmental impact, scalability, and automation.

#### 2.9.2. Continuous Synthesis of Tetrazolium

**Figure 27:**
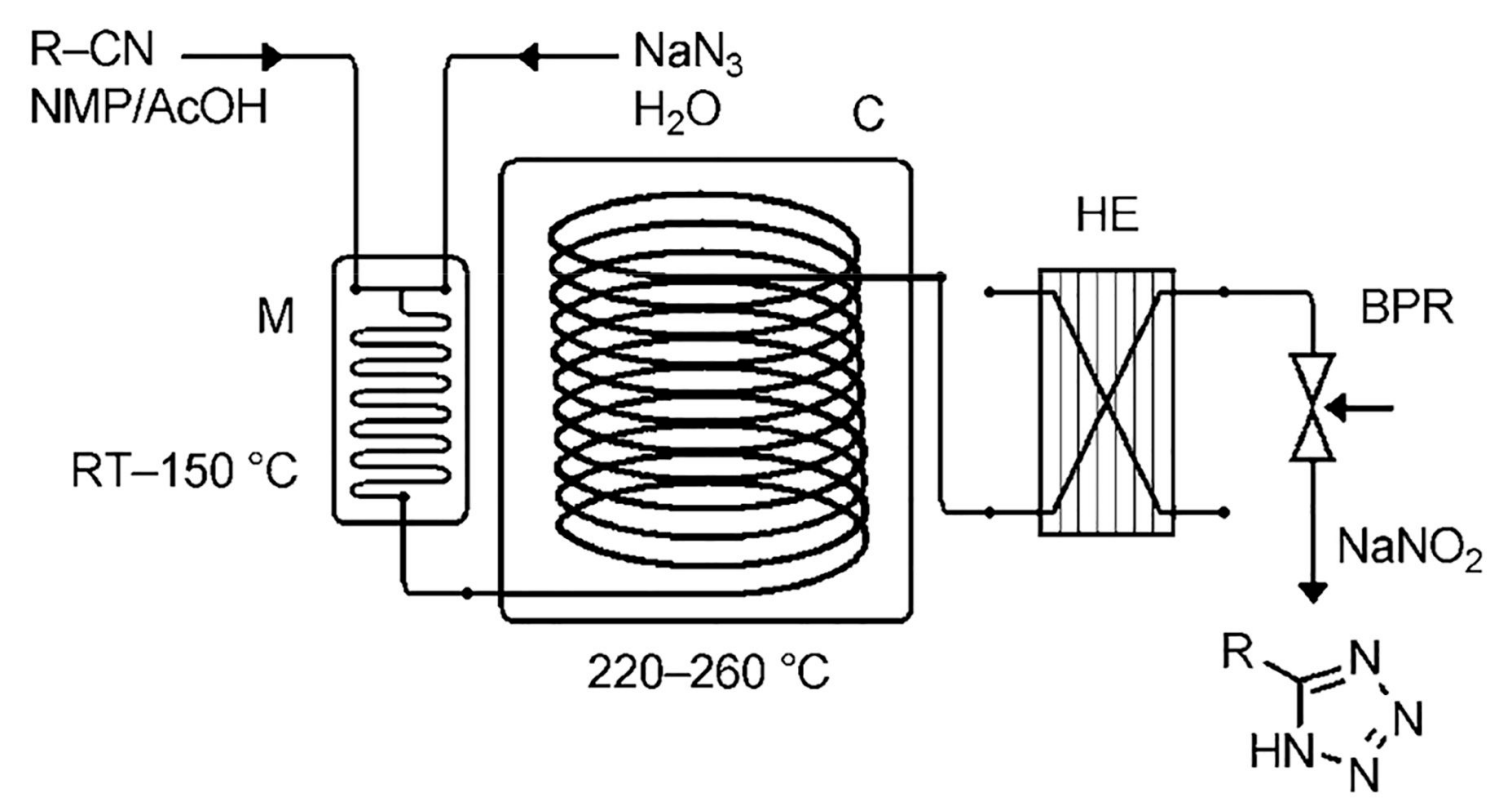
Schematic diagram for the continuous flow tetrazole synthesis performed in a FlowSyn reactor: static glass mixer block (2.0 mL internal volume); C: coil reactor (Sulfinert, 10.7 mL internal volume, 1.0 mm i.d.); HE: heat-exchanger; BPR: back pressure regulator (34 bar) [89].

In 2010, Gutman’s research group pioneered the in-situ formation of high-energy HN_3_ by mixing the inexpensive azide source NaN_3_ with acetic acid, followed by a high-temperature, high-pressure cycloaddition with organic amines to produce 1H-tetrazole without a catalyst (Figure 27). To accommodate the incompatibility of HN_3_ with metals, a passivated silica-coated stainless-steel coil (Sulfinert) reactor was used, achieving a throughput of up to 19 g/h. This cost-effective and atom-economic method is well-suited for industrial-scale tetrazole synthesis [89].

**Figure 28:**
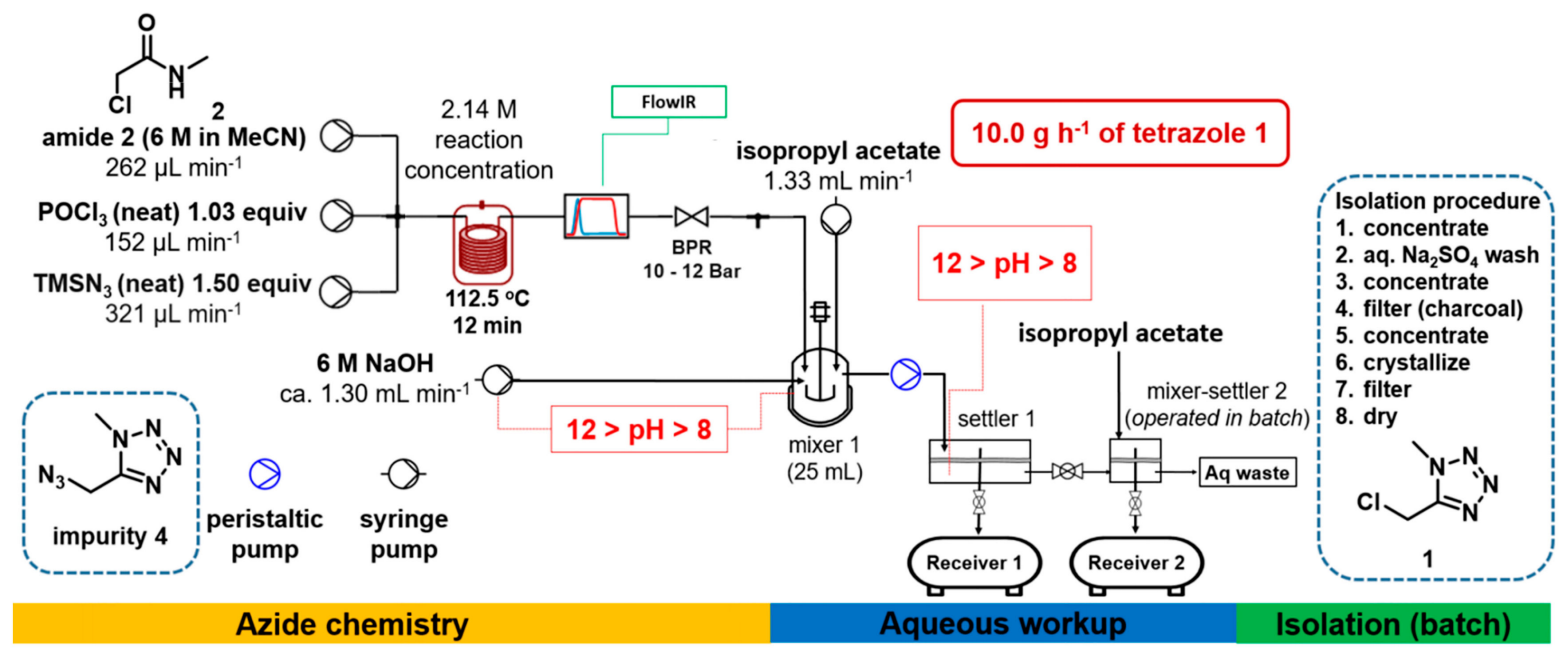
Laboratory setup for process development at Roche, including continuous aqueous quench and extraction. Product 1 was isolated in high purity following the steps outlined here. Impurity 4 was avoided by preventing the reaction of product 1 with azide anion. Reprinted with permission from Ref. [90]. Copyright 2021 Copyright American Chemical Society.

1,5-Disubstituted tetrazoles are valuable side chains in cannabinoid receptor 2 (CB2) agonists used to treat chronic pain and neurodegenerative diseases (Figure 28). The synthesis employs a three-step continuous flow process involving amide activation with POCl_3_, subsequent acyl chloride formation, and reaction with trimethylsilyl azide through diazotization and [3 + 2] cycloaddition. Full conversion was achieved within a residence time as short as 10 min, with yields of 77–86% and purities exceeding 99.7%. The continuous flow system incorporates pH-controlled quenching and extraction steps. During optimization, real-time NMR and FTIR PAT methods quantified starting materials and products, achieving a spatiotemporal yield of 1.16 kg·L^−1^·h^−1^ [90].

The above method allows for the control of reaction conditions and high yields in a short time, making it highly suitable for large-scale industrial applications. Additionally, the 1,5-tetrazole functional group was chosen due to its importance in medicinal chemistry, and the use of azide chemistry for its synthesis offers a clean and efficient pathway.

**Figure 29:**
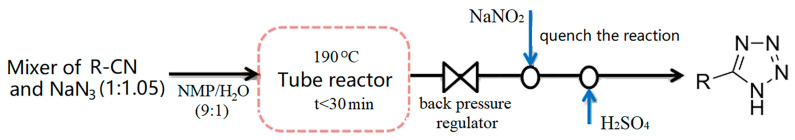
Safe and efficient tetrazole synthesis in a continuous flow microreactor [91].

Tetrazoles, crucial heterocycles in fields like organocatalysis, transition metal catalysis, propellants, and medicinal chemistry, are commonly synthesized in a continuous flow microreactor. NaN_3_ dissolved in water and added to an NMP-dissolved nitro compound flows through a heated PFA tube reactor (190 °C). After completion, the tetrazole product undergoes acidification and extraction (Figure 29). This method minimizes the need for impact-sensitive metal azides (e.g., Zn (N_3_)_2_) and enables online quenching of residual NaN_3_ with NaNO_2_. With careful optimization of solvents and reaction conditions, this approach achieves high yields quickly and safely. This technology is low-cost, simple to assemble, and suitable for both laboratory and production scale, with yields over 90% [91]. Gutmann et al. developed rapid high-temperature conversion reactions using hydrazine hydrate in a closed microwave system to achieve high-purity products quickly. However, due to the explosiveness of HN_3_, these batch processes are unsuitable for large-scale reactions. Continuous flow in microstructured reactors mitigates this risk. Microreactors offer small volume, high pressure, and temperature capabilities, which are advantageous for high-temperature or volatile/explosive reagent reactions. HN_3_ concentrations in microreactors can be safely maintained at higher levels than in sealed microwave containers due to the absence of dead space, significantly reducing explosion risk [92]. The method allows for the control of reaction conditions and high yields in a short time, making it highly suitable for large-scale industrial applications. Additionally, the 1,5-tetrazole functional group was chosen due to its importance in medicinal chemistry, and the use of azide chemistry for its synthesis offers a clean and efficient pathway.

**Scheme 5:**
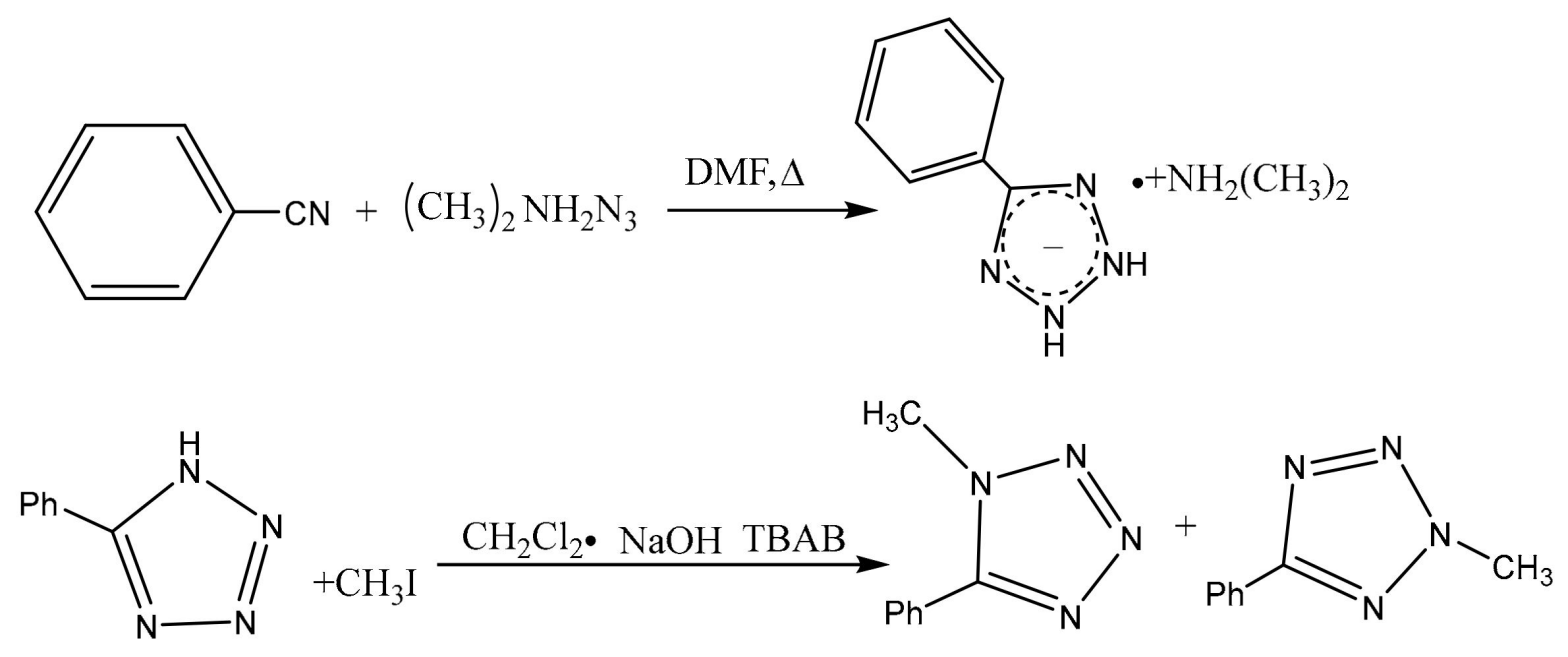
Scheme of benzonitrile azidation and 5-phenyltetrazole methylation [93].

Popova et al. explored a microreactor approach for synthesizing 5R tetrazole via aziridination, leveraging advantages, such as compact design, efficient heat and mass transfer, high reaction selectivity, and effective temperature control. Using dimethylamino nitrate as the aziridination reagent, the researchers determined the reaction rate constant for benzonitrile, finding it comparable to batch reactors (Scheme 5). Alkylation of 5-phenyltetrazole with methyl iodide was studied under various conditions, including with/without phase transfer catalysts. Increased pressure in microreactors notably accelerated the reaction rate compared to batch reactors. This method safely synthesizes NH-unsubstituted and N-substituted 5R tetrazoles [93]. The authors chose microreactor conditions for their ability to accelerate reactions and provide better control over reaction conditions, such as temperature and pressure.

**Figure 30:**
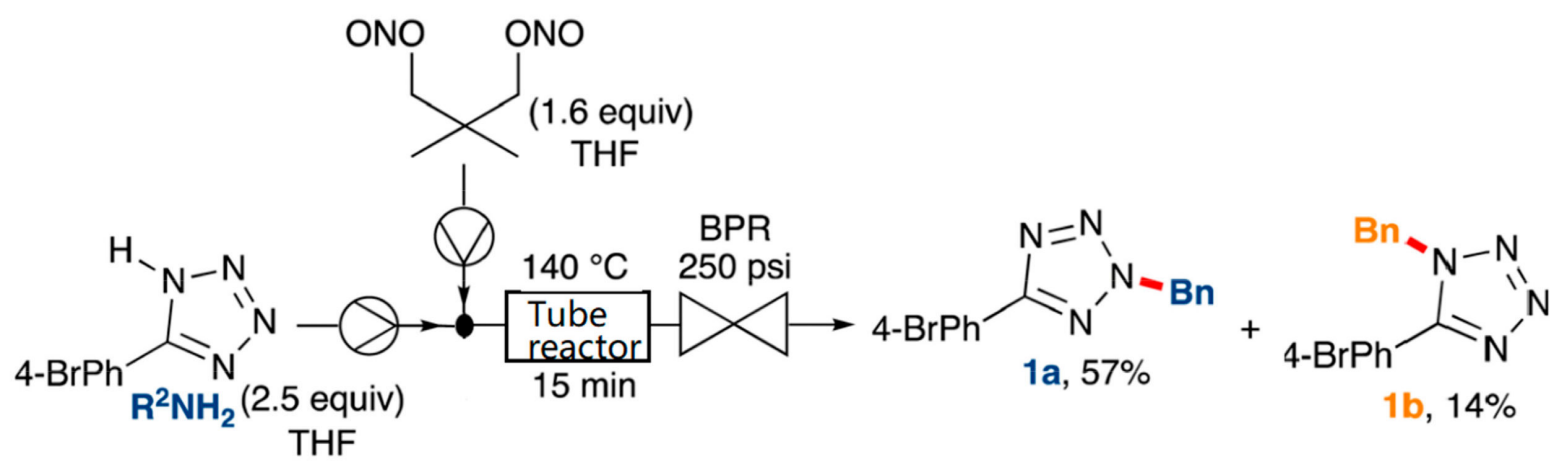
Continuous flow alkylation of 1H-Tz via amine diazotization [94].

Reynard et al. described a continuous synthesis method for alkylating tetrazole via amine diazotization to produce 2,5-disubstituted tetrazole (2,5-Tz) (Figure 30). The regioselectivity of products (1,5-Tz:2,5-Tz) showed considerable variation, influenced by factors beyond simple steric hindrance, including differences in nucleophilic substitution mechanisms (first-order vs. second-order). These insights enhance predictability of product selectivity based on substrates, guiding rational design in tetrazole synthesis [94]. 

The key differences lie in the methodology—microreactors versus diazotization—and the focus on reaction efficiency in [93] versus substitution patterns in [94]. Both methods use tetrazoles due to their bioactivity and importance in medicinal chemistry, but they differ in how they modify the tetrazole scaffold.

In addition to the example of continuous synthesis of HOF synthetic mentioned above, other studies are listed in Table 1.

## 3. Conclusions

The developed continuous flow system has been successfully applied to synthesize various HOF synthons heterocycles. It showed high catalytic activity and catalyst lifetime, increased turnover number (TON), and expanded substrate range. Continuous flow reactions can be carried out under “overheated” conditions, further improving reaction efficiency. Several innovative methods have proven new target compounds and highly efficient assembly methods tailored specifically to flow modes. In addition, the continuous synthesis of new chemical structures is increasingly adopting online analysis and purification technologies, which is crucial for providing high-purity samples in a more automated and efficient manner. Therefore, future efforts in this field may focus on synthons’ “polymerization degree” controllable, linking the synthesis of HOF structures with the evaluation of key biological characteristics, thus accelerating the discovery of new bioactive entities and HOF materials.

## Figures and Tables

**Table 1:** Continuous flow synthesis of HOF synthons.

HOFs Synthons	Conditions		Key Properties		Entry
	Reactants	RT (min), T (°C)	Yield (%)	Reactor	
urea	amines, alkyl isocyanates	10–15, 50–80	99	glass microreactor	[40]
3-fluoroalkyl pyrazole	diazoalkanes, CF_3_CHN_2_	56, 40–70	38,43,70	PFA or stainless steel coil	[58]
Pyrazole derivatives	methylhydrazine, 1,3-dicarbonyls	78.5, 90	94-97	fixed bed	[59]
pyrazole	lithium acetylide, hydrazines	4.71, 20	94	microtubular reactors	[95]
pyrazole	N-phenylsydnone, phenylacetylene	5, 140	95.5	packed reactor	[96]
glipizide, glibenclamide	amide, fcarbamate	10, 80	80-85	Glass microchannel	[97]
lorazepam	(2-amino-5-chlorophenyl)(2-chlorophenyl)methanone	5,20	54	PTFE tube	[98]
pyridine	aldehyde, ammonia	2.2 s, 450–500	75	fluidized bed	[69]
N-oxidation of pyridine	H_2_O_2_, pyridine	3–18,	99	packed-bed microreactor	[99]
triazoles, pyrimidines	N-formylglycine, triphosgene	26, 85	85	tube reactor	[100]
5-(aminomethyl)-2-methylpyrimidin-4-amine	2-cyanoacetamide	74	84	tube reactor	[101]
cycloalka[c]pyridines	doxazoles, cycloalkenes	120, 230	76	microchannel	[102]
5-(thiazol-2-yl)-3,4-dihydropyrimidin-2(1H)-ones	acetophenone, thioamide	15, 150	39–46	microchannel	[43]
N-cyclohexyl-2-benzothiazole sulfenamide	2-mercaptobenzothiazole, cyclohexylamine	3.3	87	microchannel	[103]
benzothiazoles	indole, amine	30, 25	33–82	PTFE tube	[104]
4-disubstituted 1,2,3-triazole derivatives	benzyl bromide, phenylacetylene, sodium azide, CuI, sodium ascorbate	2.88, 50	85–95	PTFE tube	[105]
1,2,3-triazoles	phenylazide and phenylacetylene	2.1, 110	96	Packed bed	[106]
Rufinamide	2,6-difluorobenzyl bromide, biphenyl	11, 110	92	copper tubing reactor	[107]
1-methyl-3,5-dinitro-1,2,4-triazole	1-methyl-3,5-diamine-1H-1,2,4-triazole	5, 30	80.5	PFA tube coiled reactor	[108]
1,2,3-triazole-substituted β-aminocyclohexane carboxylic acid derivatives	azide, diethyl acetylenedicarboxylate	5, 25	96	Packed bed	[109]
triazoles	azides, dipolarophiles	10, 80	95	Packed bed	[110]
N_1_-aryl-1,2,3-triazoles	alkyne, azide	2.7–20, 60	71–85	Polymer tube	[111]
5-amino-2-aryl-2H-[1,2,3]-triazole-4-carbonitriles	anilines, malononitrile	35, 25	72–98	PFA tube	[112]
N-sulfonyl-1,2,3-triazoles	azavinyl carbene	13.09, 75	92–98	tube reactor	[113]
1,2,4-triazole-3-thiol	hydrazides and isothiocyanates	48	92	tube reactor	[88]

## Data Availability

Not applicable.
